# Perspectives About Modulating Host Immune System in Targeting SARS-CoV-2 in India

**DOI:** 10.3389/fgene.2021.637362

**Published:** 2021-02-16

**Authors:** Sreyashi Majumdar, Rohit Verma, Avishek Saha, Parthasarathi Bhattacharyya, Pradipta Maji, Milan Surjit, Manikuntala Kundu, Joyoti Basu, Sudipto Saha

**Affiliations:** ^1^Division of Bioinformatics, Bose Institute, Kolkata, India; ^2^Virology Laboratory, Vaccine and Infectious Disease Research Centre, Translational Health Science and Technology Institute, NCR Biotech Science Cluster, Faridabad, India; ^3^Ubiquitous Analytical Techniques, CSIR-Central Scientific Instruments Organisation, Chandigarh, India; ^4^Department of Respiratory Medicine, Institute of Pulmocare and Research, Kolkata, India; ^5^Biomedical Imaging and Bioinformatics Lab, Machine Intelligence Unit, Indian Statistical Institute, Kolkata, India; ^6^Department of Chemistry, Bose Institute, Kolkata, India

**Keywords:** SARS-CoV-2, genetic variations, host immuno-modulation, repurposed drugs, vaccines, medicinal plants, CT scans, artificial intelligence

## Abstract

Severe acute respiratory syndrome coronavirus-2 (SARS-CoV-2), the causative agent of coronavirus induced disease-2019 (COVID-19), is a type of common cold virus responsible for a global pandemic which requires immediate measures for its containment. India has the world’s largest population aged between 10 and 40 years. At the same time, India has a large number of individuals with diabetes, hypertension and kidney diseases, who are at a high risk of developing COVID-19. A vaccine against the SARS-CoV-2, may offer immediate protection from the causative agent of COVID-19, however, the protective memory may be short-lived. Even if vaccination is broadly successful in the world, India has a large and diverse population with over one-third being below the poverty line. Therefore, the success of a vaccine, even when one becomes available, is uncertain, making it necessary to focus on alternate approaches of tackling the disease. In this review, we discuss the differences in COVID-19 death/infection ratio between urban and rural India; and the probable role of the immune system, co-morbidities and associated nutritional status in dictating the death rate of COVID-19 patients in rural and urban India. Also, we focus on strategies for developing masks, vaccines, diagnostics and the role of drugs targeting host-virus protein-protein interactions in enhancing host immunity. We also discuss India’s strengths including the resources of medicinal plants, good food habits and the role of information technology in combating COVID-19. We focus on the Government of India’s measures and strategies for creating awareness in the containment of COVID-19 infection across the country.

## Introduction

Coronavirus disease 2019 (COVID-19) outbreak, caused by the novel coronavirus (SARS-CoV-2) has emerged as a global epidemic and posed serious worldwide public health concerns owing to the contagious nature of the virus and high death rate. Transmission through droplets facilitated its rapid spread and caused panic across the globe. There were 80,776,890 confirmed cases worldwide till December 30, 2020^1^. India has also been largely affected by instances of COVID-19. SARS-CoV-2 viral protein interacts with various host proteins to mediate viral entry and replication in the human host (Khorsand et al., 2020). Targeting virus and host protein-protein interactions or downstream signaling cascades using novel or repositioned drugs, serves as one of the strategies for COVID-19 therapy. Several drugs such as remdesivir, dexamethasone, hydroxychloroquine, ivermectin, azithromycin, tocalizumab, famotidine, thalidomide have been evaluated in different countries for their efficacy in treating COVID-19 (Omolo et al., 2020). Convalescent plasma therapy has been recommended by FDA as an alternative therapeutic strategy for severe forms of COVID-19 infection^2^. Vaccination has been considered as the major option for containing the COVID-19 pandemic. Presently, 172 vaccine candidates are in developmental stage, while 63 have entered clinical trials^3^. The Oxford COVID-19 group have clinically proven the safety of the ChAdOx1 nCoV-19 vaccine in triggering humoral and cellular immune response against SARS-CoV-2. The vaccine is presently under the phase 3 trial program across the world (Folegatti et al., 2020). The phase 3 trials of Covishield, the Oxford vaccine in India have been conducted under the supervision of Serum Institute of India, Pune and the vaccine has been approved for emergency supply and use in India^4^^,5^. COVAXIN has been developed as India’s first indigenous vaccine by Bharat Biotech in association with Indian Council of Medical Research (ICMR). COVAXIN has currently gone into Phase III clinical trial after successful completion of Phase I and II clinical trials started by Bharat Biotech from July, 2020 onwards^6^. Recently, the Drug Controller General of India (DCGI) has granted emergency approval to COVAXIN in India^7^. The Ministry of Ayush under the Govt. of India has emphasized the importance of exploiting medicinal herbs in the context of COVID-19. Indian indigenous medicinal plants with immune regulating properties have often served to boost immunity and render protection against viral infections (Akram et al., 2018; Mohanraj et al., 2018). Besides these, the Govt. of India has adopted certain strategies such as social distancing and extensive lockdown for effective containment of COVID-19 and has launched the artificial intelligence (AI) based mobile application Aarogya Setu to create public awareness.

Computational bioinformatics and AI have been exploited for better management of COVID-19. Machine learning techniques (MLTs) have been employed for taxonomic and hierarchical classification of SARS-CoV-2 strains (Randhawa et al., 2020). Computational approaches used in CRISPR based detection systems and neural network for COVID-19 detection have increased diagnostic accuracy (Alimadadi et al., 2020; Li et al., 2020). Deep learning technology based on pulmonary CT scan images has successfully allowed differentiation of COVID-19 from other respiratory diseases such as community acquired pneumonia (Li et al., 2020). Novel text mining based collection of COVID-19 related big data, followed by subsequent analyses using advanced machine learning techniques have enabled real time surveillance of viral epidemiology and live tracking of COVID-19 cases. Access to these digital big data through mobile applications allows potential risk assessment and rapid information dissemination in public for creating social awareness and better mitigation of COVID-19 (Alimadadi et al., 2020; Bragazzi et al., 2020; Srinivasa Rao and Vazquez, 2020; Ting et al., 2020). Besides, bioinformatics tools and AI are of prime importance in drug discovery and vaccine development for SARS-CoV-2. Repurposing of existing drugs and computation based drug target identification have been extensively performed to accelerate the therapy of COVID-19. In silico docking and deep learning based drug designing have been employed to develop novel drugs against SARS-CoV-2 (Alimadadi et al., 2020; Bragazzi et al., 2020; Senior et al., 2020). A deep learning system Alphafold was designed by Google DeepMind for identification of protein structures linked with COVID-19 that might be valuable for vaccine formulation (Senior et al., 2020). Vaxign reverse vaccinology tool amalgamated with machine learning has also been used to predict vaccine candidates for COVID-19 (Ong et al., 2020). B-cell epitopes and MHC Class II epitopes can also be predicted using bioinformatics tools for peptide based vaccine development (Jabbari and Rezaei, 2019). Potential vaccine adjuvants can also be screened using the AI based program named Search Algorithm for Ligands (SAM) (Ahuja et al., 2020).

**FIGURE 1:**
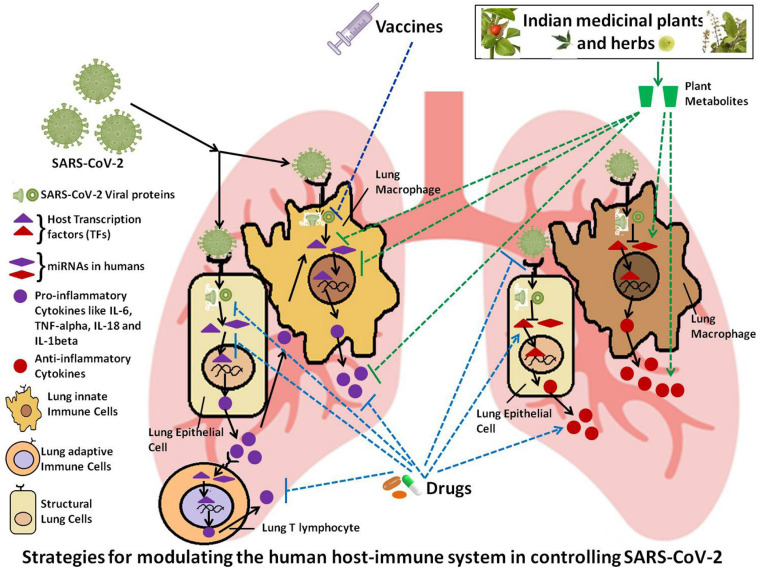
Schematic representation of the possible strategies for modulating the host immune system in order to control SARS-CoV-2 infection.

In a nutshell, this review highlights the current scenario of COVID-19 across India, with special emphasis on death/infection ratio in urban and rural India and disease association with co-morbidities. This review also deals with strains of SARS-CoV-2 circulating in India and the immuno-modulatory action of viral proteins. It discusses the various diagnostic kits, masks and disinfection techniques in use for diagnosing and combating COVID-19. This review further focuses on various approaches that may be followed to tackle the problem of SARS-CoV-2 infection (summarized in Figure 1), including immuno-regulating drugs, drugs targeting host-viral protein-protein interactions, vaccines and Indian herbs and plants with medicinal and immuno-modulating properties. Lastly, it deals with role of AI and various Government strategies adopted in India for addressing the COVID-19 pandemic.

## General Scenario in India

### COVID-19 Infection and Death Rate in States and Union territories (UTs) of India: Association With Lifestyle Habits, Proximity to Airport and Urbanization

**FIGURE 2:**
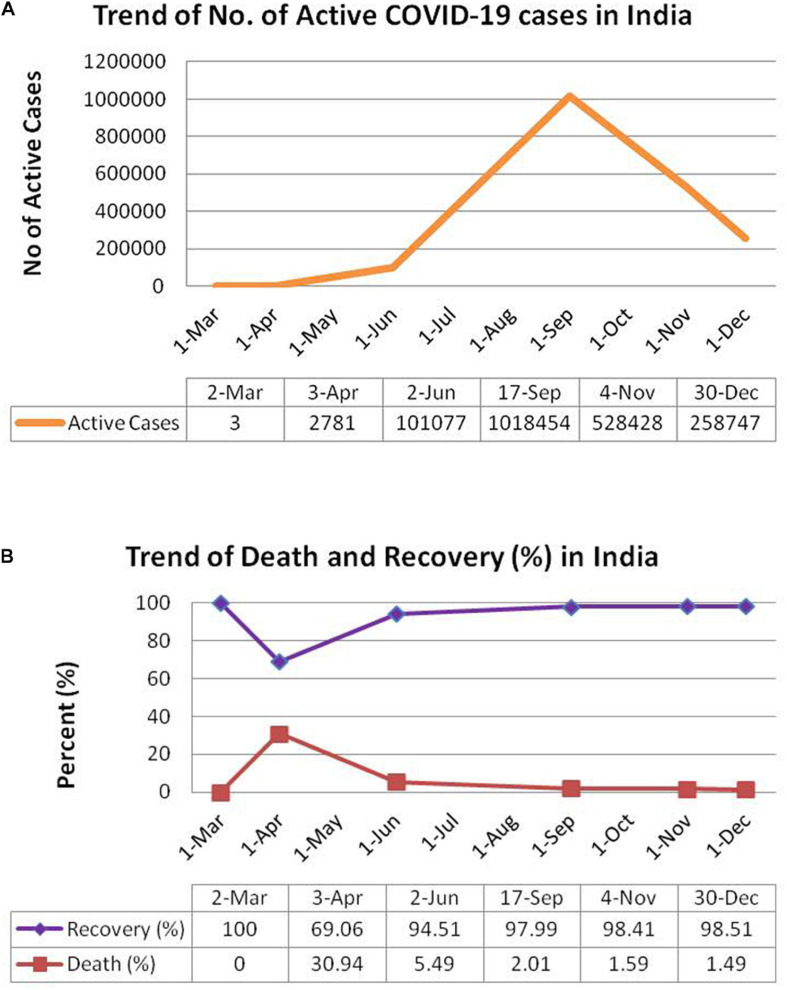
Schematic representation of trend of COVID-19 pandemic in India. **(A)** Total no. of active cases in India. **(B)** Percentage death and recovery in India. Panels **(A)** and **(B)** have been adopted from https://www.worldometers.info/coronavirus/country/india/.

The first case of COVID-19 in India was diagnosed in March, 2020. From that time onwards, there has been rise in the incidence of COVID-19 in India with 2,871 and 101,077 active cases on April 3, 2020 and June 2, 2020 respectively. The number of active cases reached a peak of 1,018,454 on September 17, 2020 followed by a decrease to 528,428 active cases on November 4, 2020 and 258,747 active cases on December 30, 2020 and (as evident from Figure 2A). Till December 30, 2020, there has been a total of 10,267,283 confirmed cases in India with a total of 148,774 deaths^8^. In terms of total number of cases, India occupies the second position after United States and is followed by developed nations such as Brazil, Russia, France, and the United Kingdom (as shown in Supplementary Table 1). The numbers of daily new cases have reduced considerably in December, 2020 as compared to that in September, 2020. There has been a consistent increase in percent recovery from April to December with a minimum recovery rate of 69.06% in April 3, 2020 to a recovery rate of 98.51% on December 30, 2020 (as shown in Figure 2B). Likewise, the death percentage has declined to 1.49% on December 30, 2020 after a surge of 30.94% in April 3, 2020 (see text footnote 71). The decrease in COVID-19 deaths (in terms of death/total confirmed case ratio) across different states of India from June, 2020 to December, 2020 has been tabulated in Supplementary Table 2.

States and cities of India harboring busy international airports (such as Kolkata in West Bengal; Ahmedabad, Surat in Gujrat; Mumbai in Maharashtra; New Delhi in Delhi and Chennai in Tamil Nadu) have shown high COVID-19 death rates. The total number of confirmed cases and death rates in Indian cities with important international airports has been tabulated in Supplementary Table 3.

**FIGURE 3:**
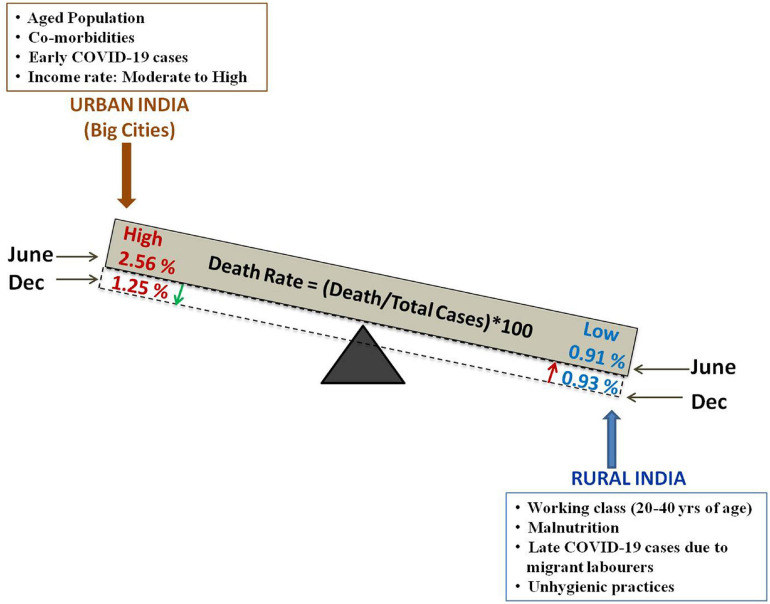
Schematic representation of the difference in death rate [expressed as (Death/total confirmed case) × 100] in the urban and rural Indian population along with the probable factors contributing to the variation in death rate (%). The green downward arrow denotes decrease death rate (%) in urban India and the red upward arrow denotes slight increase death rate (%) in rural India.

The incidences of COVID-19, death/total cases ratio and death rate have been higher in urban India than in rural areas. The death rate in urban India showed a decline in November, 2020 but, there is no significant change in the rural COVID-19 death rate (as evident from Figure 3, Table 1 and Supplementary Table 4). Early COVID-19 cases in India were primarily diagnosed in cities. Subsequent to the movement of migrant laborers from urban to rural areas and easing of transportation between rural and urban areas, there has been an increase in COVID-19 cases in rural India. High population density, greater economic activity, infrastructure development and movement of people contribute to constraints in social distancing in urban areas. Urban food habits (fast food, alcohol consumption), lifestyle patterns (improper sleep pattern, lack of physical exercise, stress) and high levels of pollution result in non-communicable lifestyle diseases (such as obesity, diabetes, and hypertension), which create additional complications in COVID-19 patients. Instances of such disorders are lower among rural population. Besides, rural lifestyle practices such as consumption of hot food, prolonged periods of sun exposure due to agricultural field work, lesser crowding, limited instances of handshaking may prove to be advantageous in conferring protection from COVID-19 (Mishra S. et al., 2020). Correlation analyses carried out in rural and semi rural areas indicate very weak positive correlation of COVID Fatality Rate (CFR) and hypertension; mild negative correlation of CFR with diabetes, implying that CFR is not necessarily related to co-morbidities such as hypertension and diabetes in rural areas^9^. Although, reduced to some extent by the Swachh Bharat Mission, a large proportion of rural households avail open defecation and public toilet facilities. Also, many rural households travel long distances to carry drinking water from community source. Social distancing becomes a difficult proposition in such situations (Mishra S. V. et al., 2020). Further, many rural households do not have exclusive rooms for individuals, thus making self isolation difficult. So, careful monitoring of urban-rural movement and augmentation of rural healthcare facilities, wherever necessary, is required to control rise in rural COVID-19 cases and death rates.

**TABLE 1:** Percentage death rate in urban and rural population across some states of India.

Sl.No	States	Urban or rural	Districts considered for study	Death rate (in %) (as on 10.06.2020)	Death rate (in %) (as on 04.11.2020)	Death rate (in %) (as on 30.12.2020)
1.	West Bengal	Urban	Kolkata, North 24 Parganas, South 24 Parganas, Howrah, Hooghly	4.45	2.24	2.15
		Rural	Malda, Paschim Medinipur, Purba Medinipur, Nadia, Puruliya	0.91	1.08	1.15
2.	Odisha	Urban	Khordha, Nayagarh, Cuttack, Puri, Malkangiri	0.44	0.55	0.64
		Rural	Ganjam, Balangir, Debagarh, Mayurbhanj, Jagatsinghapur	0.12	0.51	0.62
3.	Bihar	Urban	Patna, Gaya, Nalanda, Bhagalpur, Bengusarai	0.56	0.68	0.70
		Rural	Samastipur, Banka, Madhubani, Kaimur, Madhepura	0.31	0.47	0.50
4.	Uttar Pradesh	Urban	Lucknow, Ghaziabad, Agra, Meerut, Kanpur Nagar	4.14	1.75	1.60
		Rural	Allahabad, Azamgarh, Jaunpur, Sitapur, Gorakhpur	2.22	1.50	1.53
5.	Jharkhand	Urban	Ranchi, Dhanbad, Bokaro, Purbi Singhbhum, Ramgarh	1.95	1.08	1.12
		Rural	Giridih, Palamu, Hazaribagh, Pashchimi Singhbhum, Simdega	0.38	0.52	0.57
6.	Madhya Pradesh	Urban	Bhopal, Indore, Gwalior, Jabalpur, Ujjain	4.21	1.91	1.61
		Rural	Dindori, Jhabua, Bhind, Morena, Rewa	2.74	0.75	0.73
7.	Haryana	Urban	Gurgaon, Panipat, Faridabad, Rohtak, Sonipat	2.12	0.87	0.92
		Rural	Palwal, Rewari, Mewat, Jhajjar, Fatehabad	0	1.19	1.41

*Death rate = (Death/total confirmed case) × 100; Death rate computed with data obtained on 10.06.2020, 04.11.2020 and 30.12.2020 https://Bing.Com/Covid/Local/India.*

### Association of COVID-19 With Other Co-morbidities in India

Globally, common co-morbidities such as hypertension, diabetes, asthma, cardiovascular disease (CVD), chronic obstructive pulmonary disease (COPD), obesity, chronic kidney disease (CKD), cerebro-vascular accident (CVA), malignancy, and inflammatory conditions have been noted to worsen health status in COVID-19 positive patients (Renu et al., 2020). Medications used in these conditions often lead to upregulation of Angiotensin-converting enzyme 2 (ACE−2) receptor; thereby enhancing the possibility of ACE2 mediated viral entry and susceptibility to SARS-CoV-2 infection (Shahid et al., 2020). Communicable diseases such as tuberculosis and HIV-AIDS (Human immunodeficiency virus – Acquired Immuno Deficiency Syndrome) have also been associated with escalated severity and death rate in COVID-19 patients across the world. Sporadic studies from different Indian states/cities such as West Bengal and Jaipur revealed association of one or more co-morbid conditions with deaths in COVID-19 patients. Computational analysis based on Boolean search highlighted diabetes as the most prevalent co-morbidity in Indian COVID-19 patients, followed by hypertension (Singh and Misra, 2020). Co-morbidities in COVID-19 patients result in increased medical complications, incidence of hospitalization and high mortality rate. In order to deal with medical complications arising from COVID-19, it is vital to have knowledge regarding the SARS-CoV-2 strains and the viral mode of action within the host system.

### SARS-CoV-2 Strains Available in India and Their Evolution

Phylogenetic studies denote the causative agent of COVID-19 as belonging to the family *Coronaviridae*. Viruses belonging to this family have a single-stranded, (+) sense RNA genome of ∼30 kb (Yadav et al., 2020). During the 18th to 19th centuries, viruses from these families were known to cause infections only in animals (Cui et al., 2019). The first time it was discovered in humans was in mid 1965. This strain was referred as HCoV 229E in the United States. This was followed by an outbreak of coronavirus in France caused by another member of the same family, HCoV OC43 that led to 501 confirmed cases in 2000–2001. Till date seven different coronaviruses have been identified in this family that cause infection in humans. There have been five subsequent outbreaks in two decades prior to the recent pandemic caused by SARS-CoV-2 in December 2019 that originated from Wuhan city, China. Bioinformatics based analyses on SARS-CoV-2 genomes isolated from different countries shows its close relation with two bat origin SARS-CoV (bat-SL-CoVZC45 and bat-SL-CoVZXC21). Further, in-depth analysis of SARS-CoV-2 sequence exhibits 96.3% genome similarity with Bat CoV RaTG13 (Yadav et al., 2020). Upon comparison of SARS-CoV-2 with SARS-CoV, six different mutations were identified in ORF1a/b, S, ORF7b, and ORF8 genes. Moreover, similarity between RdRp and 3CLPro proteins has been reported. ORF8 and ORF10 show no homology with that of SARS-CoV strain (Kaur et al., 2020). Till now, no confirmed animal reservoir has been identified, although pangolins are claimed to be natural reservoirs due to the high similarity of the spike region between human SARS-CoV-2 and pangolin SARS-CoV (Andersen et al., 2020). Viruses belonging to this family have an anomalous feature of rapid mutation in their genome that causes variability in the strain. Studies are being conducted to understand the genetic diversity and evolution to establish a reference sequence for SARS-CoV-2 through mathematical modeling and Single Nucleotide Polymorphism (SNP) analysis of all the available sequences (Wang et al., 2020b). In the context of therapeutic drug and vaccine development, it is essential to monitor and track local and global genetic variations in the genome (Yin, 2020). A study of 3636 SARS-CoV-2 RNA sequences from 55 different countries revealed a remarkable mutation in the S protein at D614 amino acid position (D614G) among all the high-frequency mutations and was classified as A2a subtype. These high-frequency mutations in the SARS COV-2 genome have resulted in 11 different clusters of related sequences. Among these, O type is an ancestral type that arose from China. SARS-CoV-2 genotypes A, B, C have been described previously. These have been further divided into subtypes B, B1, B2, and B4 on the basis of mutations in the ORF8 region of SARS CoV-2. Genotype A possesses a mutation that is carried by all the B2 subtypes. A1a, a subtype of A, possesses a mutation similar to type C that may merge all these previously reported genetic variations in one cluster. There is inadequate information about the A2a subtype of SARS-CoV-2 that had spread widely in March. The A2a genotype of SARS CoV-2 consists of a non-synonymous mutation located near the S1-S2 junction similar to the A2 subtype. This non-synonymous mutation could possibly impact viral entry into the host cell (Biswas and Majumder, 2020). Thus, A2a variants could be important genetic variants for the development of effective vaccines and drugs against this virus. Further, sub-genotypes A3, A7, A1a, A2, and A6 have evolved from genotype A due to variation at the ORF1a, ORF3a, S, and nucleotide T514C respectively (Samaddar et al., 2020). Another group in India has examined 591 different novel coronaviruses and grouped them in five different clades. A total of 43% synonymous and 57% non-synonymous nucleotide substitutions were observed. The maximum number of non-synonymous substitutions was observed in the S protein (Saha et al., 2020). The presence of four SNPs at genomic positions 241, 3037, 144410, 23405 among 50–60% of the novel coronavirus population was deciphered by combining different bioinformatics (Tiwari and Mishra, 2020). However, epidemiological studies undertaken from time to time and surveillance of genetic variants among humans as well as in animals could be a major aid in the management of such outbreaks.

## Viral Mode of Action: Immuno-Modulatory Action of Viral Proteins

Binding of SARS-CoV-2 spike (S) protein with host cell angiotensin-converting enzyme 2 (ACE2) aided by TMPRSS2 mediates viral entry. SARS-CoV-2 viral proteins (enlisted in Table 2 and Supplementary Table 5) modulate host immune system and antagonize IFN response. COVID-19 pathophysiology is associated with aggressive pro-inflammatory responses (including IL-6, IL-1β, IP-10, macrophage inflammatory protein 1α (MIP1α), MIP1β and MCP1) and airway damage. Disease severity depends on viral load and the host immune response. Severe COVID-19 patients exhibit high level of pro-inflammatory macrophages, neutrophils and monocytes, which contribute to the cytokine storm with very high plasma levels of TNF, IL-12, IL-6, IL-10, IL-7, G-CSF, IP-10, MIP1α, and MCP1 (Chen et al., 2020; Liao et al., 2020; Zhou et al., 2020). Vigorous pro-inflammatory response leads to airway epithelial and endothelial cell apoptosis, respiratory microvascular damage, vascular leakage and edema, thereby causing hypoxia and compromising blood gas exchange, resulting in acute respiratory distress syndrome (ARDS) (Ye et al., 2020; Zhang B. et al., 2020). Activation of complement pathways has been associated with microvascular injury and thrombosis in severe COVID infection (Magro et al., 2020).

**TABLE 2:** Comparative list of SARS-CoV and SARS-CoV-2 viral proteins involved in modulating the host anti-viral immune response.

Sl.No.	Viral protein	Category	Host immuno modulating function of viral proteins	
			**SARS-CoV**	**SARS-CoV-2**
1.	M protein	Structural Protein (Important component of viral envelope)	Increased M protein expression is linked with RIG-I, TBK1, IKKε, and TRAF3 and hence, prevention of IRF3 and IRF7 activation. This results in significant decrease in induction of the interferon-β promoter by dsRNA (Weiss and Leibowitz, 2011).	–
2.	N protein	Structural Protein (Encodes for Nucleocapsid protein)	Overexpression is associated with decreased IFN response via inhibition of IRF3 and NF-κB responsive promoter mediated activation (Weiss and Leibowitz, 2011).	–
3.	Nsp1	Non-Structural Protein	Suppresses the activation of IRF3, c-Jun and NF-κB, thereby blocking the interferon response and subsequent activation of interferon-dependent anti-viral proteins (such as ISG15 and ISG56) (Weiss and Leibowitz, 2011).	Modulates and suppresses the host anti-viral immune response (Gordon et al., 2020).
4.	Nsp3	Non-Structural Protein	Serves as papain like protease with de-ubiquitinating activity; could act as Type I Interferon antagonist (Weiss and Leibowitz, 2011).	–
5.	Nsp13	Non-Structural Protein	–	Targets innate immune pathways such as Interferon (IFN) and NF-κB pathways (Azkur et al., 2020).
6.	Nsp15	Non-Structural Protein	–	Targets the Interferon (IFN) pathway (Azkur et al., 2020).
7.	ORF3a	Accessory Protein	Raises level of fibrinogen in lungs.	Activates the NLRP3 inflammasome (Gordon et al., 2020).
			Activates the NLRP3 inflammasome (Chen et al., 2019).	
				Activates caspase-1.
			Activates NF-κB and JNK which in turn leads to upregulated expression of pro-inflammatory cytokines (such as IL8 and RANTES) (Narayanan et al., 2008).	
				Mediates IL-1β and IL-18 secretion (Chen et al., 2019; Gordon et al., 2020).
			Induces increased apoptosis via caspase 8 and caspase 9-mediated pathways. Bax, p53 and p38 MAP kinase are also involved in ORF3a mediated apoptosis (McBride and Fielding, 2012; Chen et al., 2019).	
8.	ORF3b	Accessory Protein	Enhances the production of cytokines and chemokines by regulating the transcriptional activity of RUNX1b. Inhibits Type I interferon (IFN) production and signaling (Narayanan et al., 2008; McBride and Fielding, 2012).	IFN antagonist; regulates IRF3 activity (Gordon et al., 2020).
9.	ORF6	Accessory Protein	Promotes DNA synthesis.	Serves as a Type I Interferon (IFN) antagonist (Gordon et al., 2020).
			Hampers Type I IFN production and signaling (Narayanan et al., 2008; McBride and Fielding, 2012).	
10.	ORF7a	Accessory Protein	Triggers inflammatory response through activation of NF-κB and IL8 promoter region (Narayanan et al., 2008).	Mediates virus induced apoptosis (Gordon et al., 2020).
			Promotes pro-inflammatory cytokines (such as IL8 and RANTES) production (McBride and Fielding, 2012).	
11.	ORF8b	Accessory Protein	Blocks the IFN-β signaling pathway by ubiquitin-proteasome mediated degradation of IRF3 (Wong et al., 2018).	–
12.	Orf9b	Accessory Protein	–	Serves as a Type I Interferon (IFN) antagonist (Azkur et al., 2020).
13.	Orf9c	Accessory Protein	–	Targets the NF-κB pathway and hence the anti-viral innate immune response (Azkur et al., 2020).

## Diagnostic Methods, Therapeutic Strategies and Government Initiatives to Combat COVID-19 in India

### COVID-19 Diagnosis in India

Similarity in signs and symptoms with other respiratory infectious diseases (fever, chills, cough, and shortness of breath) put an extra burden on specialized COVID-19 diagnosis (Kaushik et al., 2020). Clinical manifestation of COVID 19 patients vary day to day and asymptomatic carriers of SARS-CoV-2 pose a challenge to our diagnostic approaches. ICMR and WHO have categorized COVID-19 as mild, moderate, and severe^10^ (Sivasankarapillai et al., 2020). Accurate and rapid diagnosis is needed to minimize substantial morbidity and mortality. Virus isolation, electron microscopy, genomic sequencing-the standard procedures for coronavirus diagnosis are time-consuming and costly. Thus, to examine a large number of patients, serological and laboratory-based methods such as CBC, AST, ALT, creatinine, LDH, ferritin examination, and molecular-based assays are being used on priority (Balachandar et al., 2020). India has set up several diagnostic and ilabs all over the country to test COVID-19 patients on the basis of qRT-PCR (Kaushik et al., 2020; Lamba, 2020). Diagnosis depends on several SARS-CoV-2 proteins, namely, spike (S), M, envelope (E), N, RdRp and ORF-1b-nsp14 (Alagarasu et al., 2020; Mourya et al., 2020). Initially, in India the first two SARS-COV-2 viruses were identified and confirmed by screening for viral genes (E, RdRp, and N protein of SARS-CoV-2) in 881 suspected cases by RT-PCR and next-generation sequencing (Yadav et al., 2020). The limited supply of positive controls has been overcome by the introduction of *in vitro* transcribed RNA from the National Institute of Virology (NIV) (Choudhary et al., 2020). SOPs for types of specimen collection and transportation were initially documented by ICMR-NIV (Gupta et al., 2020). To enhance the speed of detection, various rapid detection kits, CT scan and X-ray based techniques have been introduced from time to time. However, lack of accuracy of these techniques has prevented them from being used as standard procedures (Iyer et al., 2020). The production of IgG and IgM against COVID-19 takes 10–15 days from infection. This is a limitation for any antigen and antibody-based rapid detection kit (Hou et al., 2020). Recently, a CRISPR based fast and highly accurate diagnostic approach for COVID-19 has been introduced which employs nucleic acid readout of SARS-COV-2 (Lotfi and Rezaei, 2020). However, its implementation is highly challenging. CSIR-Institute of Genomics and Integrative Biology (CSIR-IGIB), India has also developed an efficient and accurate detection tool named Feluda based on CRISPR-Cas9 technology, as an alternative to current gold standard RT-PCR technique. Feluda has received approval from the DCGI for commercial launch^11^. In continuing efforts to discover a fast and rapid detection technique for SARS-CoV-2, an aptamer based assay has been developed at Translational Health Science and Technology Institute (THSTI). In this assay, nasal swab is used as the specimen for detection^12^. Gargle lavage sample collected from COVID-19 patients was identified as an easy, alternative showing comparable efficiency as nasopharyngeal and oropharyngeal swab samples (Mittal et al., 2020). Monitoring of patients before and after recovery through epidemiological and immunological assays in the large cohort would help understanding the prognosis and pathogenesis of COVID-19 and shall also aid in preventing community transmission and post recovery complications.

#### Detection Equipment

Standard diagnosis for infection requires real-time thermal cyclers which are used to perform RT-PCR, a robust and reliable detection technology (Corman et al., 2020). Technology centres under MSMEs began manufacturing components of Real Time Quantitative Micro PCR System in order to assemble the devices at a manufacturing unit in Visakhapatnam to ramp up the testing procedure^13^. Apart from RT-PCR based testing, other approaches have also been demonstrated which involve two-step detection methods involving more affordable thermal cyclers (conventional PCR) and fluorescence spectrometers^14^.

### The Treatment of SARS-CoV-2 Infection and COVID-19: The Present Scenario

The SARS-CoV-2 infection and the COVID-19 pandemic have posed an unprecedented challenge to the medical fraternity. The treatment is restricted to the best supportive care and experimental medications. Targeting the viral entry, interaction of the virus with its host and the downstream signaling pathways using novel or repurposed drugs, is one of the strategies for the management of COVID-19. Several agents (enlisted in Table 3 and Supplementary Table 6) have been tried based on their role in similar viral infections, or their prospective action on the novel corona virus.

**TABLE 3:** List of immuno-modulating drugs which are tried for COVID-19.

Sl.No.	Name of drugs	Type of anti viral or immune boosting action
1.	Chloroquine and Hydroxychloroquine (HCQS)	May keep the virus out of host cells by blocking host receptor glycosylation or by breaking down viral protein production.
		May lead to suppression of pH-dependent steps of viral replication (Choudhary and Sharma, 2020).
		May exert immune-modulatory effects by inhibiting TNF-α and IL6 production and may serve as a potent autophagy inhibitor.
		Active against SARS-CoV-2 in vitro (Yao et al., 2020).
		Leads to fast symptomatic improvement (fever, cough and chest imaging) (Cortegiani et al., 2020)
		HCQS and azithromycin combination leads to early viral clearance compared to HCQS alone (Gautret et al., 2020).
		US-FDA have cautioned against the use of HCQS for COVID-19 outside hospital settings https://Medicaldialogues.In/Medicine/News/Fda-Cautions-against-Use-of-Chloroquine-or-Hcqs-in-Covid-19-65165 (accessed on May 5.2020)..
2.	Corticosteroids	Exert immune-modulatory effects by inhibiting expression of genes encoding inflammatory molecules (Shaffer, 2020).
		Dexamethasone proven to be a life saving drug for severe COVID-19.
3.	Tocilizumab, Sarilumab and Situximab	Monoclonal antibody (MAb) antagonists of the IL6 receptor.
		Drugs commonly used for treatment of rheumatoid arthritis.
		Severe forms of COVID-19 are associated with elevated levels of IL6, causing acute respiratory distress syndrome (ARDS) even upon reduction of viral load.
		These MAbs may play a vital role in reducing IL6 level and reduce instances of ARDS in COVID-19 patients (Chan et al., 2013; Luo et al., 2020; Michot et al., 2020; Shaffer, 2020).
4.	Fluvoxamine	This serotonin re-uptake inhibitor may serve as an immune modulatory agent and shut down the inflammatory cascade from the endoplasmic reticulum by binding to the sigma-1 receptor (Shaffer, 2020).
5.	Remdesivir	Antiviral pro drug.
		The active analog of the pro drug inhibits the viral RNA dependent RNA polymerase (RdRp) and preventing viral replication.
		Remdesivir also evades the proofreading mechanism (exoribonuclease) of coronavirus (Ferner and Aronson, 2020; Wang M. et al., 2020) https://Www.Fda.Gov/Media/137564/Download (downloaded on May 5, 2020)..
6.	Azithromycin	Broad spectrum macrolide antibiotic.
		Used mainly for treatment of pulmonary, enteric and genitourinarytract infections. Acts as an acidotropic lipophilic weak base which modifies the pH of the endosome and trans-Golgi network and affects viral replication.
		Interferes with viral entry by binding to viral spike (S) protein and humanreceptor protein ACE2 (angiotensin converting enzyme-2).
		May exert interferon mediated anti viral immune response (Choudhary and Sharma, 2020; Damle et al., 2020).
7.	Baricitinib, Fedratinib, and Ruxolitinib	Potent JAK inhibitors selectively inhibiting JAK-STAT signaling https://Www.Chictr.Org.Cn/Showprojen.Aspx?Proj=49088 (Spinelli et al., 2020; Stebbing et al., 2020).
		Exerts anti-inflammatory effects.
		Might be effective in controlling the cytokine storm in COVID-19.
		Baricitinib is also predicted to hamper ACE2 mediated endocytosis (Richardson et al., 2020).
8.	Gimsilumab, Lenzilumab, Namilumab	These are anti-granulocyte-macrophage colony-stimulating factor (GM-CSF) antibodies. Blocks the inflammatory pathway in its early steps.
		Being clinically tested for efficacy in COVID-19 (Tay et al., 2020).
9.	Thalidomide	Synthetic glutamic acid derivative.
		Possess anti-inflammatory, anti-fibrotic, anti-angiogenesis, and immuno-modulatory effects.
		Inhibits and downregulates COX2, PGE2, TNF-α, IL6 and IL1.
		Used to treat severe H1N1 influenza-associated lung injury.
		Being tested for its efficacy in treating cytokine storm and reducing lung injury and respiratory complications in COVID-19 https://Clinicaltrials.Gov/Ct2/Show/Nct04273529^,^ https://Clinicaltrials.Gov/Ct2/Show/Nct04273581 (Khalil et al., 2020).
10.	Nafamostat and Camostat	Serine protease inhibitors which prevent SARS-CoV-2 entry by acting as antagonists to the serine protease TMPRSS2 (Yamamoto et al., 2016; Hoffmann et al., 2020; Zhang H. et al., 2020).
11.	Famotidine	H2 receptor antagonist; may bind to SARS-CoV-2 encoded papain like protease and impair entry of SARS-CoV-2 (Shaffer, 2020).
12.	Ivermectin	Broad spectrum anti-parasitic macrolide drug.
		Functions by binding and impairing the cell transport proteins that are vital for entry into the nucleus (Choudhary and Sharma, 2020).
13.	Favipiravir	Inhibits virus replication by binding and blocking the RdRp enzyme (Furuta et al., 2013).
		Its incorporation in RNA also terminates viral protein synthesis (Jin et al., 2013).
		Classically used against influenza virus.
		Also acts on SARS-CoV-2 replication; used for mild and moderate COVID cases (Agrawal et al., 2020).
14.	Lopinavir/ Ritonavir	Antiretroviral protease inhibitors, successfully used in HIV infection (Huang et al., 2015).
		Combination of lopinavir/ritonavir used successfully for treatment of SARS with significantly fewer adverse clinical outcomes (Chu et al., 2004).
		Lopinavir/ Ritonavir with Interferon 1b found promising in the marmoset model (Chan et al., 2015).
15.	APN01	Soluble form of ACE2 delivered in high concentrations.
		Could potentially block SARS-CoV-2 entry into target cells. Under clinical trial https://Pipelinereview.Com/Index.Php/2020022673884/Proteins-and-Peptides/Apeirons-Respiratory-Drug-Product-to-Start-Pilot-Clinical-Trial-to-Treat-Coronavirus-Disease-Covid-19-in-China.html.

Indian Pharmaceuticals Cadila has tested the immunomodulator drug named Sepsivac (containing heat-killed *Mycobacterium* W (Mw)), on COVID-19 patients at PGIMER, Chandigarh in partnership with the Council of Scientific and Industrial Research (CSIR) to reduce the mortality of critically ill COVID-19 patients and have obtained promising results^15^.

Apart from these drugs, the US FDA has approved use of convalescent plasma for severe life-threatening COVID infection as an investigational new drug (Duan et al., 2020). Its use has been documented in a series of cases (Huang et al., 2020; Zeng et al., 2020)^16^. One small trial with five ventilated patients showed success. Its role is still not clear and US FDA is facilitating the use of hyperimmune globulin for COVID treatment (Mehta et al., 2020). US FDA recommended the use of convalescent plasma for emerging infections including COVID-19 on May 1, 2020 (see text footnote 2). The Indian Council of Medical Research (ICMR) began clinical trials with convalescent plasma in India to evaluate its safety and efficiency in controlling COVID-19 symptoms^17^^,18^. ICMR has recommended use of convalescent plasma for COVID-19 therapy. A plasma bank has been established in Delhi and Project PLATINA has been established in Maharashtra for treatment cum trial with plasma therapy^19^.

Another approach for developing drugs targeting host immunity has been to express SARS-CoV-2 proteins in human cell lines and identify their human protein interacting partners. Of 332 interactions, 66 human proteins were found as druggable candidates that could be targeted by 29 FDA approved drugs, 12 compounds in clinical trials and 28 compounds in preclinical stage (Gordon et al., 2020). Further screening has helped in the identification of two pharmacological candidates that inhibit mRNA translation and are predicted to regulate Sigma1 and Sigma2 receptors. Besides, inhibitors targeting endocytosis have shown activity in vitro against other coronaviruses such as SARS CoV and MERS-CoV. These include chlorpromazine, ouabain and bufalin (de Wilde et al., 2014; Burkard et al., 2015). Their efficacy against SARS CoV-2 is yet to be tested. However, very high EC_50_/C_max_ (half-maximal effective concentration value/peak serum concentration level) ratio at the typical dosages used is limiting their possible clinical use.

Natural killer cells play a role in the clearance of SARS-CoV. NK cell based products are in various stages of trial as anti-COVID-19 agents. The US-based Company Celularity has developed placenta derived NK cells CYNK-001 (Tu et al., 2020). Recombinant Interferon Type I exhibits broad spectrum activity against coronaviruses (Cinatl et al., 2003b; Sheahan et al., 2020). Clinical trials are currently in motion for the treatment of COVID-19 pneumonia (NCT04293887). Trials are also ongoing to test the efficacy of mesenchymal stem cells from the umbilical cord and dental pulp to attenuate the inflammatory response of COVID-19 (NCT04293692, NCT04269525, NCT04288102, NCT04302519). The World Health Organization’s (WHO) Solidarity trial including randomized and controlled clinical trials are set to test several protocols against COVID-19.

### COVID-19 Vaccine Developments – Present Indian Scenario

In the global fight against COVID-19, scientists from different countries are trying to decipher a potential therapeutic drug, vaccine, and early diagnostic tools. The SARS-CoV-2 ‘S’ protein interacts with the ACE2 receptor and is a glycosylated protein, making this protein a good candidate for vaccine development (Othman et al., 2020). Globally several vaccine generation methods are being used against COVID-19, including a live attenuated vaccine, inactivated vaccine, replicating viral vector, non-replicating viral vector, DNA vaccine, peptide-based vaccine, recombinant protein, virus-like particle (VLP) and mRNA-based vaccine (Le et al., 2020). According to the WHO, there are currently 63 COVID-19 vaccines in clinical development and 172 vaccine candidates in pre clinical developmental stage as on 6th January, 2021 (see text footnote 3). Out of these 63 vaccines, about 20 vaccine candidates are in Phase III clinical trial (as enlisted in Table 4). Among these 20 vaccines, the efficacy report is available for five vaccines that include “BNT162 (Pfizer), mRNA 1273 (Moderna), chAdOX1nCOV19 (University of Oxford and AstraZeneca), BBIBP-CorV (Sinopharm) and Sputnik-V (Gamaleya Research Institute)^20^. However, only three vaccines are available with the data published in peer reviewed journals till 5th January, 2021 namely, mRNA1273, BNT162, and chAdOX1nCOV19 (Baden et al., 2020; Polack et al., 2020; Voysey et al., 2020). Supporting data to answer such important question such as duration of herd immunity upon vaccination, requirement of booster doses for long term immunity and whether vaccine could help in the prevention of transmission is available for only chAdOX1nCOV19 vaccine till to date. Further, safety of the above mentioned vaccines needs to be evaluated in the populations that have not been included in the trials such as pregnant women (see text footnote 21).

**TABLE 4:** List of vaccines in Phase III trial across the world.

Sl.No.	Name of vaccine	Nature of vaccine	Clinical Trial Phase	Country of origin
1	Ad5-nCoV	Recombinant vaccine (adenovirus type 5 vector)	Phase III	CanSino Biologics Inc/Beijing Institute of Biotechnology (China)
2	Covishield (Code name: AZD1222)	Replication-deficient viral vector vaccine (adenovirus from chimpanzees)	Phase III (received approval for emergency use in United Kingdom and India)	The University of Oxford; AstraZeneca; IQVIA; Serum Institute of India (Multinational) (see text footnote 4) https://Www.Bbc.Com/News/Health-55280671
3	CoronaVac	Inactivated viral vaccine (formalin with alum adjuvant)	Phase III	Sinovac (China)
4	COVAXIN	Inactivated viral vaccine	Phase III (approved for emergency use in India)	Bharat Biotech; National Institute of Virology (India) (see text footnote 7)
5	JNJ-78436735 (formerly Ad26.COV2-S)	Non-replicating viral vector	Phase III	Johnson & Johnson (Janssen Pharmaceutical) (United States)
6	mRNA-1273	mRNA-based vaccine	Phase III (received approval and presently in use in United States)	Moderna; National Institute of Allergy and Infectious Diseases (NIAID) (United States) https://Www.Nature.Com/Articles/D41586-020-03593-7
7	New Crown COVID-19 Vaccine	Inactivated vaccine	Phase III	Wuhan Institute of Biological Products; China National Pharmaceutical Group (Sinopharm, China) https://Www.Precisionvaccinations.Com/Vaccines/New-Crown-Covid-19-Vaccine
8	NVX-CoV2373	Protein based vaccine (Full length recombinant SARS CoV-2 spike protein nanoparticle vaccine adjuvanted with Matrix M)	Phase III	Novavax (Maryland); Serum Institute of India https://Ir.Novavax.Com/News-Releases/News-Release-Details/Novavax-Announces-Covid-19-Vaccine-Clinical-Development-Progress^,^ https://Www.Verywellhealth.Com/Novavax-Covid-19-Vaccine-5093292
9	BNT162 (3 LNP-mRNAs)	mRNA-based vaccine	Phase II/III (Already in use in United Kingdom and United States)	Pfizer; BioNTech; Fosun Pharma; Jiangsu Provincial Center for Disease Prevention and Control (Multinational) https://Www.Thehindu.Com/News/International/Uk-Approves-Pfizer-Biontech-Covid-19-Vaccine-for-Use/Article33228634.Ece (https://www.nature.com/articles/d41586-020-03593-7)
10	Sputnik-V Vaccine (rAd26-S+rAd5-S)	Adeno viral vector based technology	Phase III	Gamaleya Research Institute; Health Ministry of the Russian Federation (Russia)
11	BBIBP-CorV	Inactivated viral vaccine	Phase III	Sinopharm + Beijing Institute of Biological Products (China) (Xia et al., 2021)
12		Recombinant SARS-CoV-2 vaccine	Phase III	Anhui Zhifei Longcom Biopharmaceutical; Institute of Microbiology, Chinese Academy of Sciences (China)
13	INO-4800	DNA based vaccine	Phase II/III	Inovio Pharmaceuticals and International Vaccine Institute (South Korea)
14	CoVLP	Coronavirus-Like Particle based vaccine	Phase II/III	Medicago Inc. (Canada)
15	CVnCoV	RNA based vaccine	Phase II/III	CureVac AG (Germany)
16	UB-612	Multitope peptide based S1-RBD-protein based vaccine	Phase II/III	COVAXX; United Biomedical Inc
17	ZyCoV-D nCov vaccine	DNA based vaccine	Phase III	Cadila Healthcare Ltd. Zydus Cadila, (India)https://Economictimes.Indiatimes.Com/Markets/Stocks/News/Cadila-Healthcare-Gains-3-as-Dcgi-Plays-Phase-Iii-Trials-of-Covid-Vaccine/Articleshow/80091363.Cms
18	QazCovid-in	Inactivated viral vaccine	Phase III	Research Institute for Biological Safety Problems (Rep of Kazakhstan)
19	SARS-CoV-2 vaccine (vero cell)	Inactivated viral vaccine	Phase III	Institute of Medical Biology; Chinese Academy of Medical Sciences (China)
20	AG0301-COVID19	DNA based vaccine	Phase II/III	AnGes + Takara Bio + Osaka University (Japan)

*Vaccine Information obtained from World Health Organization (WHO) as on 06.01.2021 (see text footnote 3).*

Apart from this, global mass immunization also encountered several challenges including financial, logistic and vaccine storage-related issues. The upper middle income countries started the vaccination in 2020. However, successful global vaccination or complete eradication of virus is possible only when low income and middle income countries get immunized in parallel. To overcome this situation, an International initiative termed COVAX facility has been set up to ensure equitable access to vaccine doses in Low income and middle income countries (LCMICs). COVAX aims at fixed vaccination for 20% of population belonging to the LCMICs by 2021. The vaccine will be provided by the AstraZeneca^21^.

In India, COVAXIN, an indigenous inactivated COVID-19 vaccine, stable at 2–8°C, manufactured by Bharat Biotech (Hyderabad, India) has currently entered the Phase III Human clinical trial and has recently been given emergency approval in India by the DCGI (see text footnote 7)^22^. A plasmid DNA based vaccine, ZyCoV-D has been developed by Ahmedabad-based pharma company Cadila Healthcare (Zydus Cadila). It has been claimed that this vaccine is stable for 3 months at a temperature of 30°C and longer at 2–8°C. This thermostability could be beneficial for nationwide vaccination program due to minimalistic cold storage requirements. Phase III human clinical trials are being initiated for this vaccine. Besides these indigenous vaccines, several non indigenous vaccines of foreign origin are presently in various stages of clinical trial in India. Covishield, the vaccine developed by Oxford University and AstraZeneca has entered phase III trials in collaboration with the Serum Institute, Pune, India. Serum Institute has applied to DCGI for emergency regulatory authorization for Covishield use in India and has submitted additional requisite vaccine datasheet in this regard. This vaccine has been approved for emergency use in United Kingdom and has become the first COVID-19 vaccine candidate to have obtained emergency approval in India (see text footnote 4)^23^. Covishield has the advantage of storage at 2–8°C^24^. Besides this, Dr. Reddy’s Laboratories Limited and Sputnik LLC (Russia) have been jointly conducting the clinical trial of Sputnik-V, the world’s first registered vaccine in India. This vaccine, ranking among world’s top 10 vaccine candidates is presently in Phase II Human Clinical Trial in India^25^. The Biological E’s novel Covid-19 vaccine is also in the Phase I/II Human Clinical Trial in India (see text footnote 5). Ecological studies have highlighted lower number of infections and reduced COVID-19 mortality in countries, where BCG vaccination is made mandatory (Urashima et al., 2020). Randomized controlled trials of BCG-Danish have been conducted in Netherlands and Australia (NCT04327206, NCT04328441). Serum Institute, Pune, India has conducted phase III trial of BCG vaccine VPM1002 to evaluate cross-protection to COVID-19^26^. BCG vaccine could serve as a booster of innate immunity against COVID-19 via metabolic and epigenetic changes in a process called trained immunity (Netea et al., 2020).

Another important vaccine, BNT162 from Pfizer, which has already been rolled out in United Kingdom, United States and received emergency use approval in more than 10 countries, has extreme cold chain and storage requirement at −75°C to keep its potency intact^27^. Similarly, Moderna vaccine also has stringent storage requirement at −20°C^28^. Such stringent refrigeration needs may be difficult to achieve in developing countries and may render mass vaccination in India extremely challenging. Although India has cold storage facilities, they are limited and there are constraints especially in the case of handling large numbers of doses in a country as densely populated as India. Ramping up of cold chain and restructuring of cold storage facilities with synergistic aid from food storage and supply cold chain, may aid in overcoming vaccine storage issues to some extent^29^. Another important factor involved in mass vaccination is the economic burden. The COVAX facility (led by WHO, CEPI, and GAVI) have emerged to financially support and enable equitable distribution of COVID-19 vaccines across the world^30^. The Government of India (GOI) has also taken initiatives to bear the entire cost of vaccination and ensure mass immunization at nominal price^31^. The Global Alliance for Vaccines and Immunizations (GAVI) has estimated an expenditure of $1.4 billion to $1.8 billion on part of India (the second most populous country after China) for the first phase vaccination, even after support from the COVAX facility^32^. Moderna vaccine, apart from its cold requirement, is highly priced (at $10–450 per dose), which might be difficult to cater to the Indian population. However, aid from COVAX alliance and Government may help Moderna reduce its cost for India. Covishield and COVAXIN are reasonably priced with respect to the Indian scenario. Covishield have been priced at $3 per dose for government and approximately, $10 for private entities. Because of their local origin and normal refrigeration temperatures, these two vaccines will be easy for handling and supply chain distribution in India^33^^,34^.

COVID-19 mass vaccination drive in India shall soon be initiated with 30 crore people receiving the vaccines in the first phase. Healthcare workers, frontline workers and individuals aged above 50 will be vaccinated first according to the recommendations of the National Expert Group on Vaccine Administration for COVID-19 (NEGVAC). In this regard, the COVID Vaccine Intelligence Network (Co-WIN) system has been developed as a digital platform for registration and real time monitoring of vaccination to pre registered individuals in India^35^.

### Neutralizing Antibodies: Another Approach

Neutralization of the virus by antibodies is an important strategy for containing SARS-CoV-2. In SARS-CoV, the RBD122 (amino acids 318 to 510) of the S protein is primarily being targeted by neutralizing antibodies (Wong et al., 2004). The RBD of SARS-CoV and SARS CoV-2 are poorly conserved, so the majority of the monoclonal antibodies to SARS-CoV do not bind with or neutralize SARS CoV-2 (Wang et al., 2020a). Therapeutic monoclonal antibodies to SARS CoV-2 are being developed with the aid of phage library display, cloning of human B cell sequences from recovering patients and mouse immunization and hybridoma isolation. Anaïve semi synthetic library has been used to identify the anti-SARS-CoV-2 RBD human monoclonal antibody. This approach holds promise since the entire RBD remains conserved as of now (Parray et al., 2020). However, caution must be exercised, since animal studies of SARS CoV infection show that neutralizing antibodies to S protein may increase lung injury by aggravating inflammatory responses (Liu et al., 2019). Anti-S-IgG mediated proinflammatory responses occur due to binding of virus-anti-S-IgG complex with the Fc receptors (FcR) present on monocytes and macrophages (Liu et al., 2019). In addition, virus-anti-S-IgG complex may trigger the classical complement pathway leading to cellular damage.

### Indian Government Initiatives and Strategies to Combat COVID-19

#### Personal Protective Equipment

Personal Protective Equipment (PPE) including face piece respirators, gloves, shoe covers and face shields are necessary for the protection of health workers from infection^36^. N95 respirators, surgical masks or cloth masks are recommended to prevent respiratory transmission. Cloth masks may possibly be cost-effective in preventing community transmission in densely populated Asian countries (Sra et al., 2020). Unlike N95 respirators, simple cloth and surgical masks are non-disposable and can be potentially decontaminated routinely using alcohol/detergent washing, and moist heat treatment (Viscusi et al., 2009). To prevent contact transmission, disposable gloves are recommended for patient examination. Government of India is funding enterprises and enabling transfer of advanced technology for increased PPE production^37^. However, supply of raw materials may be dependent on import and could be a bottle neck for large scale production in India (Feinmann, 2020).

#### Disinfection Instruments

COVID-19 may potentially remain transmissible on inanimate surfaces up to several days. Effective disinfection could be achieved using biocidal chemicals such as 70% ethanol, 0.1% aqueous sodium hypochlorite and 0.5% hydrogen peroxide solutions (Kampf, 2020; Kampf et al., 2020). 60–70% ethanol is recommended for sterilizing high-end biomedical equipment, while 0.1% aqueous sodium hypochlorite could be a viable solution for decontamination of large areas such as mass transit systems, hospital outdoors etc. Scientists from the Council of Scientific and Industrial Research (CSIR), India have claimed to develop a spraying procedure by using induction charged electrostatic spraying apparatus involving lower amounts of chemicals, charge based disinfection and large coverage in comparison with conventional high-volume sprayers^38^^,39^ (Lyons et al., 2011). In line with other countries, drone-based disinfection methods have been proposed by Indian enterprises^40^. Concern about the potential hazards of inhaling the aerosolized disinfectants still poses a challenge for large area disinfection (Kim et al., 2020).

#### Biomedical Equipment

Various medical equipment such as ventilators, sensor equipments including pulse-oximeter, infrared thermometer, multi parametric photo plethysmography (PPG) sensor, portable X-ray machine, fiberoptic bronchoscopes, video laryngoscopes, are required in monitoring and treatment of COVID-19 patients (Wax and Christian, 2020). The ventilator is a crucial equipment for critically ill patients with respiratory problems (Iyengar et al., 2020). Ventilators are costly (∼$30,000) and there is a world-wide shortage of ventilators during the pandemic. India alone has a requirement for tens of thousands of ventilators^41^. There is global endeavor to enhance production, lower cost and find alternatives. Engineers from Rail Coach Factory have claimed production of a low-cost prototype ventilator^42^. Scientists at the CSIR laboratories are also developing 3D printed automatic ventilators and mechanical ventilators^43^ (Iyengar et al., 2020). An alternative for the ventilator, “Artificial Manual Breathing Unit (AMBU)” has been designed by researchers from the Postgraduate Institute of Medical Education and Research, Chandigarh (Iyengar et al., 2020). Recently, an Indian manufacturer has reported production of state-of-the-art ventilator costing less than $2000 (Agrawal, 2020).

#### Indigenous Medicinal Plants for Combating COVID-19

Antiviral herbal therapy has made enormous progress in the past decade (Dhama et al., 2018). Various medicinal plants and bioactive phyto-metabolites have been widely explored for effective control of several viral diseases such as influenza, hepatitis, human immunodeficiency virus (HIV), herpes simplex virus (HSV) and coxsackievirus infections (Akram et al., 2018). India harbors a diverse variety of medicinal plants and herbs with therapeutic potential (Mohanraj et al., 2018). The major indigenous medicinal plants with immuno-modulatory properties, which can potentially be explored for their role in boosting immunity and rendering protection from SARS-CoV-2 infection, have been summarized in Table 5 and Supplementary Table 7.

**TABLE 5:** List of medicinal plants with major immune modulating properties.

Sl.No.	NAME OF PLANTS	Type of anti viral or immune targeting effects exerted
1.	Turmeric	Curcumin in turmeric is an immune-modulatory agent.
		Has an anti-viral, anti-microbial, anti-inflammatory and anti-oxidant activity.
		Reduces pro-inflammatory cytokines like TNF-α, IFN-γ, IL-1 and IL-8 via interaction with signal transducers such as NF-κB, JAKs/STATSs, MAPKs and β-catenin (Lelli et al., 2017; Kahkhaie et al., 2019).
2.	Ashwagandha	Activates immune response.
		Triggers Th1 cytokines and interferon expression.
		Increases expression of co-stimulatory molecules and integrins (Khan et al., 2009).
3.	Cinnamon	Inhibits allergen specific immune responses.
		Protects from systemic inflammation and lung injury by attenuating NLRP3 inflammasome activation (Sharma et al., 2016; Xu et al., 2019; Ose et al., 2020).
4.	Cardamom	Has anti-microbial activity (Agnihotri and Wakode, 2010).
		Exerts anti-inflammatory effect by inhibiting mediators such as COX2, TNF-α and IL-6 (Majdalawieh and Carr, 2010; Kandikattu et al., 2017).
5.	Holy Basil	Exerts anti-inflammatory effects by modulating cellular and humoral immunity.
		Elevates IFN-γ and IL-4.
		Increases percentages of T-helper cells and NK-cells (Mondal et al., 2011; Kamyab and Eshraghian, 2013).
6.	Cumin	Thymoquinone in cumin has immuno-modulatory and anti-inflammatory properties. Suppresses inflammation by downregulation of COX2, IL-6, TNF-α and NO production, and enhancement of IL-10 production.
		Modulates cellular and humoral immunity and regulates Th1/Th2 immune response. Enhances NK cell mediated cytotoxicity (Majdalawieh and Fayyad, 2015; Gholamnezhad et al., 2016, 2019).
7.	Neem	Has anti-inflammatory, antibacterial and antioxidant effects.
		Attenuates release of pro-inflammatory cytokines such as TNF-α and IL-6, thus modulating immune response; inhibits MCP-1 (monocyte chemoattractant protein-1) expression and recruitment of inflammatory cells (Hao et al., 2014; Lee et al., 2017).
8.	Saffron	Has anti-inflammatory, radical scavenging and immuno-modulatory properties (Bolhassani et al., 2014; Moshiri et al., 2015).
9.	Amlaki	Has anti-inflammatory and immune-regulating activities.
		Promotes NK cell function and Antibody-dependent cellular cytotoxicity (ADCC) (Yang and Liu, 2014).
10.	Brahmi	Has immunomodulatory effectsHttp://Nopr.Niscair.Res.In/Handle/123456789/41986.
		Mediates anti inflammatory effects by preventing the release of pro-inflammatory cytokines such as IL6 and TNF-α from microglial cells and the immune cells of the brain (Nemetchek et al., 2017).
11.	Moringa	Activates CD8^+^ T cells, promotes IL-10, IL-2, IL-6 and TNF-α production (Coriolano et al., 2018).
12.	Liquorice Root (Yashtimadhu)	Glycyrrhizin, the active compound of the liquorice root, inhibits SARS-associated coronavirus replication (Cinatl et al., 2003a).
		Reduces virus uptake by host cells (especially in case of influenza virus) (Mousa, 2017).
		Glycyrrhizin also stimulates IFN-γ production by T cells.
		Exerts anti-inflammatory effects by inhibiting iNOS, COX2, IL-1β, TNF-α, IL-5 and IL-6 or by blocking trans-activation of NF-κB (Kuang et al., 2018; Fouladi et al., 2019).
13.	Shatavari	Modulates the Th1/Th2 balance; promotes IgG secretion and IL-12 production; and inhibits IL-6 production (Pise et al., 2015).
14.	Coriander	Has anti-inflammatory activity and boosts immunity (Li et al., 2016).
15.	Kapikacchu (Velvet Beans)	Modulates immune mediators such as NF-κB, IL-6, IFN-λ, TNF-α, IL-1β, iNOS and IL-2 in the central nervous system (Rai et al., 2017).
		Boosts the innate immune response (Saiyad Musthafa et al., 2018).
16.	Ajwain	Acts as an anti-inflammatory agent and exerts bronchodilatory effect (Boskabady et al., 2005, 2007; Bairwa et al., 2012).
17.	Manjishtha	Serves as potential anti-inflammatory agent and immune modulator.
		Increases functions of the lymphatic system Http://Www.Ijtsrd.Com/Papers/Ijtsrd9616.Pdf (Shen et al., 2018).
18.	Bibhitaki	Boosts immunity https://Www.Netmeds.Com/Health-Library/Post/Bibhitaki-5-Ways-This-Traditional-Fruit-Boosts-Your-Immunity.
19.	Guduchi, Giloy (Tinospora)	Serves as anti-oxidant and anti-inflammatory agent.
		Regulates NF- êB signaling and production of pro-inflammatory mediators (Dhama et al., 2017; Haque et al., 2017).
20.	Haritaki	Possesses anti-inflammatory and wound healing properties (Ratha and Joshi, 2013).
21.	Cinchona Bark	Source of chloroquine, a common anti-malarial drug; exerts an effect on SAR CoV-2 by immune modulation and blockage of viral entry (Lentini et al., 2020).
22.	Shatapushpa (Fennel)	Suppresses the immune response (Darzi et al., 2018).
		Regulates Th17 and Treg immune response (Zhang et al., 2018).
23.	Triphala	Exerts an anti-inflammatory effect via decreased expression of inflammatory mediators such as IL-17, COX-2, iNOS, TNF-α, IL-1β, VEGF, IL-6 and RANKL by preventing NF-κB activation (Peterson et al., 2017).
24.	Jatiphala (Nutmeg)	Has immuno-modulatory functions.
		Macelignan in nutmeg has anti-inflammatory property and inhibits Th2 cytokines such as IL-4 (Shin et al., 2013).
25.	Jatamansi	Has anti-inflammatory, immuno-modulatory and wound-healing properties (Pandey et al., 2013; Han et al., 2017).
26.	Vidanga	Ameliorates pro-inflammatory cytokines and suppresses TNF-α production (Shirole et al., 2015).
27.	Gokshura (Tribulus)	Can reduce inflammation and fibrosis in the lungs by lowering the expression of IL-6, IL-8, TNF-α and TGFβ1 (Qiu et al., 2019).
28.	Bhringaraj (Eclipta)	Exerts anti-inflammatory effects by regulating the NF-κB pathway and the production of pro-inflammatory mediators (Feng et al., 2019).
29.	Punarnava (Boerhavia)	Has anti-inflammatory properties (Mishra et al., 2014).
		Punarnavine, an alkaloid in Boerhavia exerts immuno-modulatory activities by reducing TNF-α, IL-1β, IL-6 production, and by increasing the titer of circulating antibody (Manu and Kuttan, 2009).
30.	Bhunimba (Andrographis)	Has anti-inflammatory and immuno-modulatory effects (Islam et al., 2018).
31.	Shankha pushpi (Dwarf morning glory)	Possesses anti-bacterial and immuno-modulating activity (Nguyen et al., 2016).
32.	Vidari (Indian Kudzu)	Serves as an immune booster and an anti-inflammatory agent by inhibiting inflammatory mediators such as CRP, NF-κB, COX-2, iNOS, TNF-α, IL-1β and IL-6 (Maji et al., 2014).

The Ministry of Ayush under the Govt. of India has recommended use of indigenous herbal plants and spices namely, tulsi, cinnamon, dry ginger, black pepper, turmeric, coriander, cumin and garlic for enhancing immunity^44^. Besides, the Ministry of Ayush has formulated a collection of four ayurvedic herbs namely, ashwagandha, guduchi, yasthimadhu, peepli; and a drug named Ayush 64 for combating COVID-19. The Ministry of Ayush along with the CSIR have initiated the process of validating the efficacy of these formulations against COVID-19 in the month of May, 2020 and the outcomes of these trials are expected to be available soon^45^^,46^^,47^.

#### Artificial Intelligence in Combating COVID-19

The worldwide outbreak of SARS-CoV-2 has resulted in a tremendous dearth of clinical equipment. In order to contain the pandemic effectively, large scale testing and diagnosis are required. This is evident from the successful containment of SARS-CoV-2 virus in countries that have been able to perform mass testing of possibly infected people and contact tracing. RT-PCR serves as the gold standard test for validating SARS-CoV-2 infection. Inadequate testing capability in most countries, along with the high dependency of the RT-PCR test on the swab technique, has spurred the need to search for alternative methods that allow COVID-19 diagnosis.

##### CT scan in COVID-19

The chest X-ray and thoracic computed tomography (CT) are examples of easily accessible medical imaging equipment, which assists clinicians in diagnosis. CT images may serve as a visual indicator of coronavirus infection for radiologists (Duncan and Ayache, 2000). While RT-PCR may take up to 24 h and needs multiple tests for conclusive results, chest CT combined with certain health symptoms can be used as an effective diagnostic tool in clinical practice for rapid screening of COVID-19 patients. There is a high chance that COVID-19 patients can be diagnosed accurately by using chest radiography images (van Ginneken et al., 2001; Sluimer et al., 2006). However, manual examination of CT scans for COVID-19 diagnosis is a labor-intensive and time-taking process. Besides, clinical presentation of COVID-19 in CT images is similar to other forms of viral pneumonia, which makes diagnosis even more difficult. A dependable computer-aided diagnostic system for COVID-19 may have huge implication in clinical practice for improving the detection efficiency while alleviating the radiologist’s workload (Dong et al., 2020; Shi et al., 2020). COVID-19 lesions in CT scans have a wide range of presentation in terms of appearance, size, and location in lungs, so, developing a system using either classical image processing approaches or conventional machine learning techniques relying on handcrafted features, is a challenging task. Recently, artificial intelligence (AI) has shown promise. It warrants better safety, higher accuracy and efficacy in imaging compared to the traditional, laborious imaging workflows. Alongside pioneering the basic clinical research, AI have enormous application in recent COVID-19 scenario which include provision for well allocated imaging platform, segmentation of infected and unaffected regions of lungs, clinical evaluation and diagnosis (Wang J. et al., 2020; Wang X. et al., 2020).

##### Role of deep learning

Deep learning technology which lies central to current concept of Artificial Intelligence has been effective in automated detection of lung diseases with high diagnostic accuracy. However, there are challenges when developing AI-empowered deep learning technologies for COVID-19 screening (Oh et al., 2020; Roy et al., 2020). Most of the deep learning based methods require annotating the lesions in CT volumes for effective disease detection. Annotating lesions and labeling of annotations are laborious and time consuming, and hence, are not desirable in times of rapid COVID-19 outbreak and simultaneous shortage of radiologists. Therefore, the major challenge of AI-empowered solutions is to determine the potential of a deep learning model based on patients’ chest CT volumes for automated and accurate COVID-19 diagnosis. It should require nominal expert annotation and should be easily trained, which will be extremely advantageous in developing AI solution rapidly for COVID-19 diagnosis. Due to the constraints of hardware resources, a major challenge is to educate a deep learning model using volumetric CT scans. Another problem is the inter-class similarity and variation across pneumonia lesions. Finally, the lung CT scan images from patients with pneumonia harbor large portions of non-lesion regions, which exhibit wide range of complex tissue level variations. These non-lesion regions often exert a negative impact on the overall performance of AI-based solutions.

#### Mobile Applications and Social Distancing Strategies

Aarogya Setu has been developed as a digital mobile COVID-19 tracking application, by the National Informatics Centre under the initiative of the Ministry of Electronics and Information Technology, Govt. of India, for effective awareness, management and mitigation of COVID-19 (Kodali et al., 2020)^48^^,49^. The Delhi Government has also launched the Delhi Corona app to create public awareness regarding availability of hospital facilities for COVID-19 treatment and also for complaint redressal regarding refusal to admit COVID-19 patients by hospitals with available facilities^50^^,51^^,52^. Apart from these mobile applications, the Govt. of India has promoted strict social distancing to contain spread of COVID-19 amongst the Indian population (Paital et al., 2020).

## Discussion

The COVID-19 health crisis has created a stir in the whole world including in India. There has been a global endeavor in terms of disease diagnosis, drug repurposing and vaccine development to combat this pandemic. In addition to actively participating in these efforts to improve therapeutics and vaccine development against SARS-CoV-2; the Government of India has taken several initiatives and measures to further contain the disease. The total numbers of active cases in India reached a peak in the month of September, 2020 and have reduced subsequently. Although there have been 148,774 deaths in India till December 30, 2020; the recovery rate of COVID-19 patients in India has increased to about 98.51% as on December 30, 2020 (see text footnote 8). COVID-19 infection may exert detrimental long term effects on organs such as lungs, liver, kidney, brain. and heart (Heneka et al., 2020). These may even last after recovery from COVID-19 and lead to life-threatening health issues^53^. Several clinical parameters such as blood levels of inflammatory mediators, neutrophil to lymphocyte ratio (NLR) and CT scan severity score have been evaluated for highlighting disease progression and the risk for development of post recovery complications such as pulmonary fibrosis, ARDS, neurological disorder or even multi-organ failure (Feng et al., 2020). Identification of blood borne easily detectable biomarkers could potentially stratify COVID-19 based on its severity and enable early prediction of progression to post recovery complications, thereby leading to better post COVID care and effective control of deaths due to such complications.

## Author Contributions

All the authors reviewed the literature, wrote the review, edited, and approved the manuscript.

## Conflict of Interest

The authors declare that the research was conducted in the absence of any commercial or financial relationships that could be construed as a potential conflict of interest.

## Footnotes

1 Covid19.Who.Int/

2 https://www.fda.gov/Vaccines-Blood-Biologics/Investigational-New-Drug-Ind-or-Device-Exemption-Ide-Process-Cber/Recommendations-Investigational-Covid-19-Convalescent-Plasma

3 https://www.who.int/Publications/M/Item/Draft-Landscape-of-Covid-19-Candidate-Vaccines

4 https://Timesofindia.Indiatimes.Com/India/Oxford-Covid-Vaccine-Set-to-Become-First-to-Get-Approval-in-India-Report/Articleshow/80059745.Cms

5 https://Vaccine.Icmr.Org.In/Covid-19-Vaccine

6 https://www.bharatbiotech.com/covaxin.html

7 https://www.expresspharma.in/covid19-updates/dcgi-approves-covishield-and-covaxin-for-restricted-emergency-use-in-india/

8 https://Www.Worldometers.Info/Coronavirus/Country/India/

9 https://Www.Thehindu.Com/Data/Data-Lower-Covid-19-Fatality-Rate-in-Rural-Areas-Not-Necessarily-Due-to-Lower-Share-of-Co-Morbidities-among-Rural-Population/Article32620632.Ece

10 https://www.expresshealthcare.in/covid19-updates/icmr-revises-treatment-protocol-for-covid-19-patients/421792/

11 https://Science.Thewire.In/the-Sciences/Explained-Feluda-Covid-19-Test-India-Crispr-Technology/

12 https://Journosdiary.Com/2020/07/12/Aptamer-Based-Assay-Developed-for-Coronavirus-Detection/

13 https://Pib.Gov.In/Pressreleasepage.Aspx?Prid=1623027

14 https://Www.Hindustantimes.Com/India-News/Iisc-Comes-up-with-an-Affordable-Two-Step-Method-to-Scale-up-Rt-Pcr-Testing/Story-Xbztkyjilgeldprouohp6o.html

15 https://Www.Hindustantimes.Com/Health/Indian-Trials-on-Multiple-Covid-19-Drugs-Make-Progress-Have-Atmanirbhar-Bharat-Tilt/Story-Nk0owrrrsyragqhvrk2a9i.html

16 https://www.fda.gov/news-events/press-announcements/coronavirus-covid-19-update-fda-coordinates-national-effort-develop-blood-related-therapies-covid-19

17 https://Www.Indialegallive.Com/Special-Story/Convalescent-Plasma-Therapy-a-Treatment-for-Covid-19/

18 https://Www.Medrxiv.Org/Content/10.1101/2020.09.03.20187252v2

19 https://Swachhindia.Ndtv.Com/Coronavirus-Outbreak-Explained-What-Is-Convalescent-Plasma-Therapy-and-How-Effective-Is-It-in-Treating-Covid-19-Patients-46568/

20 https://Www.Thelancet.Com/Journals/Lanmic/Article/Piis2666-5247(20)30226-3/Fulltext

21 https://Www.Thelancet.Com/Pdfs/Journals/Lanmic/Piis2666-5247(20)30226-3.Pdf

22 https://Www.Indiatoday.In/News-Analysis/Story/Why-Covaxin-Covishield-Best-Option-for-India-against-Covid19-Pandemic-1755517-2021-01-03

23 https://Www.Thehindu.Com/Sci-Tech/Health/Uk-Becomes-First-Country-to-Approve-Astrazeneca-Oxford-Vaccine-for-Covid-19/Article33451081.Ece

24 https://Www.Businesstoday.In/Coronavirus/Oxford-Serum-Institute-Vaccine-Stored-Fridge-Temperature-2-8-Degrees/Story/422741.html

25 https://sputnikvaccine.com/about-vaccine/

26 https://health.economictimes.indiatimes.com/news/pharma/serum-institute-conducting-phase-iii-clinical-trial-of-tuberculosis-vaccine-dbt/77210270

27 https://www.pfizer.com/news/hot-topics/covid_19_vaccine_u_s_distribution_fact_sheet

28 https://indianexpress.com/article/explained/covid-19-vaccine-storage-optimal-temperature-cold-chain-india-explained-quixplained-7063369/

29 https://Health.Economictimes.Indiatimes.Com/News/Pharma/Ramping-up-Cold-Storage-Facilities-Critical-as-India-Preps-for-Covid-19-Vaccine-Experts/78550153

30 https://Www.Who.Int/Emergencies/Diseases/Novel-Coronavirus-2019/Covid-19-Vaccines

31 https://Www.Livemint.Com/Budget/News/Govt-Will-Bear-the-Entire-Cost-of-Covid-19-Vaccination-Guleria-11608563339012.html

32 https://Science.Thewire.In/Health/India-Covid-19-Vaccine-First-Phase-1-8-Billion/

33 https://indianexpress.com/article/explained/coronavirus-vaccines-india-covishield-bharat-biotech-covaxin-7131057/

34 https://Timesofindia.Indiatimes.Com/Life-Style/Health-Fitness/Health-News/Coronavirus-Vaccine-Can-India-Get-Its-Hands-on-Modernas-Covid-19-Vaccine-3-Challenges-We-Have/Photostory/79411299.Cms?Picid=79411390

35 https://Www.Livemint.Com/News/India/India-S-Covid-19-Vaccination-Drive-to-Start-Soon-Registrations-Details-to-Guidelines-All-You-Need-to-Know-11608363252706.html

36 https://www.health.state.mn.us/facilities/patientsafety/infectioncontrol/ppe/index.html

37 https://www.investindia.gov.in/siru/personal-protective-equipment-india-INR-7000-cr-industry-in-the-making

38 https://Www.Igi-Global.Com/Chapter/Fundamentals-of-Electrostatic-Spraying/232957

39 https://Www.Tribuneindia.Com/News/Nation/Csio-Develops-Electrostatic-Disinfection-Technology-to-Combat-Covid-78098

40 https://Pib.Gov.In/Pressreleasepage.Aspx?Prid=1620351

41 https://Www.Medrxiv.Org/Content/10.1101/2020.03.26.20044511v1.Full.Pdf

42 https://Www.Tribuneindia.Com/News/Nation/Rail-Coach-Factory-Kapurthala-Develops-Ventilator-66118

43 https://Www.Csir.Res.In/Csir-Labs-Initiatives-against-Covid-19

44 https://www.ayush.gov.in/

45 https://Newsonair.Nic.In/News?Title=Ministry-of-Ayush%2c-Csir-Working-Together-on-Validating-Four-Ayush-Formulations-against-Covid-19&Id=388575 (accessed on June 22, 2020).

46 https://timesofindia.indiatimes.com/life-style/health-fitness/home-remedies/covid-19-ministry-of-ayush-starts-clinical-trials-for-ashwagandha-and-4-other-ayurvedic-herbs-here-is-what-you-need-to-know/photostory/75692669.cms

47 https://www.hindustantimes.com/india-news/trials-for-4-ayush-formulations-against-covid-19-to-start-within-a-week-says-minister/story-XU9RsNDC3vLrFukt3gOApK.html

48 https://en.wikipedia.org/wiki/Aarogya_Setu

49 https://static.mygov.in/rest/s3fs-public/mygov_159056978751307401.pdf (retrieved on June 23, 2020).

50 https://www.ndtv.com/india-news/arvind-kejriwal-launches-delhi-corona-app-for-information-on-availability-of-hospital-beds-in-delhi-2239276

51 https://indianexpress.com/article/explained/delhi-corona-mobile-application-covid-19-6438796/

52 https://Www.Thehindu.Com/News/Cities/Delhi/Kejriwal-Launches-Delhi-Corona-App-for-Real-Time-Information-on-Availability-of-Hospital-Beds/Article31729239.Ece (retrieved on June 23, 2020).

53 https://www.Thehindu.Com/Sci-Tech/Health/the-Hindu-Explains-What-Are-the-Long-Term-Effects-of-Covid-19/Article32651206.Ece
