# Supplementary material for: Perspectives About Modulating Host Immune System in Targeting SARS-CoV-2 in India
Source: Front Genet. 2021 Feb 16;12:637362. doi: 10.3389/fgene.2021.637362 (PMC7921795; doi:10.3389/fgene.2021.637362)
Supplement: Supplementary file 1 [file Data_Sheet_1.docx]

**Perspectives about modulating host immune system in targeting SARS-CoV-2 in India**

***Supplementary Materials***

**Supplementary Tables:**

**Supplementary Table 1: Comparative analysis of COVID-19 scenario in different countries across the world in terms of total no. of cases, daily COVID-19 cases, total fatality due to COVID-19 and daily deaths.**

| **Countries** | **Total no. of COVID-19 cases (as on 30.12.2020)** | **Total no. of fatalities (as on 30.12.2020)** | **No. of daily COVID-19 cases (on the following dates)** | | | | | **No. of daily COVID-19 deaths (on the following dates)** | | | | |
| --- | --- | --- | --- | --- | --- | --- | --- | --- | --- | --- | --- | --- |
|  |  |  | **3^rd^**  **Apr, 2020** | **2^nd^ Jun, 2020** | **17^th^ Sept, 2020** | **4^th^ Nov, 2020** | **30^th^ Dec, 2020** | **3^rd^**  **Apr, 2020** | **2^nd^ Jun, 2020** | **17^th^ Sept, 2020** | **4^th^ Nov, 2020** | **30^th^ Dec, 2020** |
| **USA** | 20,217,241 | 350,780 | 33,108 | 22,586 | 47,017 | 109,069 | 234,775 | 1,287 | 1,145 | 884 | 1,210 | 3,882 |
| **India** | 10,267,283 | 148,774 | 516 | 8,821 | 96,793 | 50,465 | 21,957 | 14 | 221 | 1,174 | 704 | 299 |
| **Brazil** | 7,619,970 | 193,940 | 1,150 | 27,263 | 35,757 | 23,815 | 55,853 | 39 | 1,232 | 857 | 622 | 1,224 |
| **Russia** | 3,131,550 | 56,426 | 601 | 8,863 | 5,762 | 19,768 | 26,513 | 4 | 182 | 144 | 389 | 599 |
| **France** | 2,600,498 | 64,381 | 5,233 | 0 | 10,593 | 42,496 | 26,457 | 1,119 | 107 | 50 | 437 | 303 |
| **UK** | 2,432,888 | 72,548 | 4,032 | 1,497 | 3,395 | 25,174 | 50,023 | 734 | 237 | 21 | 492 | 981 |
| **South Africa** | 1,039,161 | 28,033 | 0 | 1,455 | 2,128 | 1,712 | 17,710 | 0 | 50 | 67 | 46 | 465 |
| **Bangladesh** | 512,496 | 7,531 | 0 | 2,911 | 1,593 | 1,517 | 1,235 | 0 | 37 | 36 | 21 | 22 |
| **China** | 87,027 | 4,634 | 19 | 4 | 9 | 17 | 24 | 4 | 0 | 0 | 0 | 0 |
| **Australia** | 28,381 | 909 | 140 | 17 | 34 | 12 | 31 | 3 | 0 | 8 | 0 | 0 |

**Supplementary Table 2: State wise distribution of COVID-19 cases and deaths in India along with details on spices, food, smoking and drinking habits.** Data on total number of confirmed cases and deaths have been collected on 02.06.2020 and 04.11.2020 08.00 IST (GMT + 5.30).

Supplementary Table 2 has been attached in MsExcel Sheet named SupplementaryTable 2.xlsx

**Supplementary Table 3: List of Indian cities harboring international airports along with details of COVID-19 cases, deaths and death/infection ratios.** Data on total number of confirmed cases and deaths have been collected on 10.06.2020 and 04.11.2020 08.00 IST (GMT + 5.30).

Supplementary Table 3 has been attached in MsExcel Sheet named SupplementaryTable 3.xlsx

**Supplementary Table 4:** **Details of COVID-19 cases, deaths and death/infection ratios in rural and urban areas for 7 Indian states (which have considerable amount of rural and urban population).** Data on total number of confirmed cases and deaths have been collected on 10.06.2020 and 04.11.2020 08.00 IST (GMT + 5.30).

Supplementary Table 4 has been attached as MsExcel Sheet named SupplementaryTable 4.xlsx

**Supplementary Table 5: List of SARS-CoV-2 viral proteins along with their target host proteins and their role in viral infection.**

| **Sl.No.** | **Category** | **Viral protein** | **Host Protein Interactors** | **Functional role** |
| --- | --- | --- | --- | --- |
| 1. | Structural Protein | Spike Protein (S) | ACE2, TMPRSS2, GOLGA7, ZDHHC5. | The viral spike protein (S) binds to host receptor Angiotensin converting enzyme (ACE2).  SARS-CoV-2 cell entry largely depends on its interaction with ACE2 and TMPRSS2. |
| 2. | Structural Protein | Envelope Protein (E) | BRD2, BRD4, CWC27, AP3B1, SLC44A2, ZC3H18. | Plays a vital role in the assembly of new viral particles. |
| 3. | Structural Protein | Membrane Protein (M) | REEP6, REEP5, YIF1A, RTN4, SLC30A9, SLC30A7, SLC25A21, SAAL1, AAR2, TARS2, STOM, PSMD8, PMPCA, PMPCB, PITRM1, INTS4, GGCX, ETFA, FAM8A1, FASTKD5, COQ8B, BZW2, ATP1B1, ATP6V1A, AASS, ACADM, ANO6, AKAP8L, TUBGCP2, TUBGCP3. | Component of viral envelope.  Involved in viral assembly. |
| 4. | Structural Protein | Nucleo-capsid Protein (N) | FAM98A, SNIP1, G3BP2, G3BP1, CSNK2B, CSNK2A2, MOV10, LARP1, PABPC1, PABPC4, RRP9, RPL36, UPF1, DDX21, RBM28. | Binding and packaging of viral RNA genome. |
| 5. | Non-Structural Protein | Nsp1 | PKP2, POLA1, POLA2, PRIM1, PRIM2, COLGAT1. | Modulates and suppresses the host anti-viral immune response. |
| 6. | Non-Structural Protein | Nsp2 | POR, WASHC4, FKBP15, SLC27A2, GIGYF2, RA1GDS1, EIF4E2. | - |
| 7. | Non-Structural Protein | Nsp3 | - | Mediates viral replication. Serves as papain like protease. |
| 8. | Non-Structural Protein | Nsp4 | IDE, ALG11, NUP210, TIMM9, TIMM29, TIMM10B, TIMM10, DNAJC11. | Mediates viral replication. |
| 9. | Non-Structural Protein | Nsp5 | HDAC2. | Acts as 3C like protease, the main viral protease. |
| 10. | Non-Structural Protein | Nsp6 | ATP13A3, ATP5MG, ATP6AP1, SIGMAR1. | Mediates viral replication. |
| 11. | Non-Structural Protein | Nsp7 | RAB18, RAB10, RAB14, RAB2A, RAB1A, RAB8A, RAB5C, RAB7A, AGPS, LMAN2, GNG5, GNB1, NDUFAF2, MTARC1, RALA, RHOA, COMT, QSOX2, MOGS, PTGES, NAT14, ACSL3, DCAKD, SELENOS, FAM162A, HS2ST1, SCCPDH, CYB5R3, CYB5B, DNAJC19, TOR1AIP1. | Forms the primase complex along with Nsp8. |
| 12. | Non-Structural Protein | Nsp8 | DDX10, HECTD1, NGDN, NOL10, AATF, SRP72, SRP54, SRP19, NARS2, CCDC86, EXOSC2, EXOSC5, EXOSC3, EXOSC8, LARP7, MEPCE, NSD2, MPHOSPH10, ATE1, SEPSECS, MRPS2, MRPS25, MRPS27, MRPS5. | Forms the primase complex along with Nsp7. |
| 13. | Non-Structural Protein | Nsp9 | FBN1, FBN2, FBLN5, MIB1, SPART, NEK9, GTF2F2, DCAF7, EIF4H, MAT2B, ZNF503, NUP88, NUP58, NUP214, NUP54, NUP62. | Binds to ssRNA. |
| 14. | Non-Structural Protein | Nsp10 | GFER, GRPEL1, AP2A2, AP2M1, ERGIC1. | Possesses Nsp16 methyl transferase activity. |
| 15. | Non-Structural Protein | Nsp11 | TBCA. | Is a short peptide. |
| 16. | Non-Structural Protein | Nsp12 | RIPK1, SLU7, PPIL3, TCF12, USP54, SBNO1, TYSND1, BCKDK, ZC3H7A, ZNF318, UBAP2, UBAP2L, AKAP8, CRTC3, LARP4B, MYCBP2, PDZD11, PLEKHA5, PRRC2B, RBM41. | Serves as the RNA polymerase. |
| 17. | Non-Structural Protein | Nsp13 | AKAP9, PRKACA, HOOK1, PRKAR2A, PRKAR2B, PDE4DIP, GCC1, GCC2, GOLGB1, GOLGA2, GOLGA3, GORASP1, FYCO1, HSBP1, USP13, MIPOL1, TLE1, TLE3, TLE5, CIT, TBK1, RDX, TBKBP1, GRIPAP1, CLIP4, JAKMIP1, C1orf50, ERC1, CENPF, CEP112, CEP135, CEP250, CEP350, CEP43, CEP68, CNTRL, NIN, NINL, PCNT, CDK5RAP2. | Acts as helicase/triphosphatase.  Targets innate immune pathways such as Interferon (IFN) and NF-κB pathways. |
| 18. | Non-Structural Protein | Nsp14 | GLA, SIRT5, IMPDH2. | Serves as 3’-5’ exonuclease. |
| 19. | Non-Structural Protein | Nsp15 | ARF6, RNF41, NUTF2. | Serves as uridine specific endoribonuclease.  Targets the Interferon (IFN) pathway. |
| 20. | Non-Structural Protein | Nsp16 | - | Acts as RNA-cap methyl transferase. |
| 21. | Accessory Protein | Orf3a | ALG5, HMOX1, VPS11, VPS39, SUN2, CLCC1, TRIM59, ARL6IP6. | Activates the NLRP3 inflammasome.  Activates Caspase-1.  Mediates IL-1β and IL-18 secretion. |
| 22. | Accessory Protein | Orf3b | STOML2. | IFN antagonist; regulates IRF3 activity |
| 23. | Accessory Protein | Orf6 | MTCH1, NUP98, RAE1. | Serves as a Type I Interferon (IFN) antagonist |
| 24. | Accessory Protein | Orf7a | MIDN1, HEATR3. | Mediates virus induced apoptosis. |
| 25. | Accessory Protein | Orf7b | - | - |
| 26. | Accessory Protein | Orf8 | EDEM3, ERLEC1, OS9, UGGT2, ERO1B, SIL1, HYOU1, NGLY1, TOR1A, FOXRED2, SDF2, CHPF, ADAMTS1, TM2D3, NPC2, LOX, PVR, COL6A1, PLOD2, FKBP10, FKBP7, GGH, IL17RA, PUSL1, PLD3, PCSK6, ADAM9, ITGB1, DNMT1, EMC1, CHPF2, ERP44, MFGE8, HS6ST2, POFUT1, NPTX1, STC2, POGLUT2, PLAT, NEU1, SMOC1, GDF15, INHBE, CISD3, PLEKHF2, POGLUT3, FBXL12. | - |
| 27. | Accessory Protein | Orf9b | MARK1, MARK2, MARK3, BAG5, DPH5, TOMM70, DCTPP1, CSDE1, CHMP2A, PTBP2, SLC9A3R1. | Serves as a Type I Interferon (IFN) antagonist. |
| 28. | Accessory Protein | Orf9c | TMEM97, ERMP1, TAPT1, PIGS, GPAA1, SLC30A6, TMED5, SCAP, BCS1L, NDFIP2, DPY19L1, F2RL1, GHITM, ABCC1, ALG8, ECSIT, ACAD9, TMEM39B,  NDUFAF1, NDUFB9, FAR2, WFS1, PIGO, RETREG3, UBXN8, NLRX1. | Targets the NF-κB pathway and hence the anti-viral innate immune response. |
| 29. | Accessory Protein | Orf10 | TIMM8B, PPT1, CUL2, ELOC, ELOB, ZYG11B, RBX1, MAP7D1, THTPA. | - |

Note: The above information has been gathered from Gordon et al., 2020 and Azkur et al., 2020.

**Supplementary Table 6: List of anti-viral and immuno-modulating drugs which are tried for COVID-19.**

| **Sl.No.** | **Name of Drugs** | **Type of anti viral or immune boosting action mediated** |
| --- | --- | --- |
| 1. | Chloroquine and Hydroxychloroquine (HCQS) | Well known anti malarial drug.  May keep the virus out of host cells by blocking host receptor glycosylation or by breaking down viral protein production.  May lead to suppression of pH-dependent steps of viral replication.  May exert immune-modulatory effects by inhibiting TNF-α and IL6 production and may serve as a potent autophagy inhibitor.  May serve as a potent autophagy inhibitor  Active against SARS-CoV-2 in vitro.  Leads to fast symptomatic improvement (fever, cough and chest imaging).  HCQS and azithromycin combination leads to early viral clearance compared to HCQS alone.  US-FDA have cautioned against the use of HCQS for COVID-19 outside hospital settings. |
| 2. | Lopinavir and Ritonavir | Antiretroviral protease inhibitors, successfully used in HIV infection.  Acts against corona viruses (SARS-CoV-1 and MERS-CoV) by inhibiting 3-chymotrypsin-like proteases.  Reduced time of viral shedding, reduced time of symptomatic recovery and hospital stay was noted in mild COVID-19 patients treated with combination of the two drugs than in patients with one drug alone.  Not effective in severe COVID-19 subjects owing to side effects like liver damage, nausea, vomiting, which further worsens the scenario.  Combination of lopinavir/ritonavir used successfully for treatment of SARS with significantly fewer adverse clinical outcomes.  Lopinavir/ Ritonavir with Interferon 1b found promising in the marmoset model. |
| 3. | Nafamostat and Camostat | Serine protease inhibitors used for pancreatitis.  Prevents SARS-CoV-2 entry by acting as antagonists to the serine protease TMPRSS2 |
| 4. | Famotidine | H2 receptor antagonist widely as heartburn medication.  May bind to SARS-CoV-2 encoded papain like protease and impair entry of SARS-CoV.  Low cost, low toxicity and bioavailablity encourages testing of this drug for COVID-19. |
| 5. | Umifenovir | Small indole derivative molecule with broad spectrum anti viral properties.  This hydrophobic molecule interacts with both lipid and proteins.  Binds to viral lipid membrane and affects cellular trafficking of virus, thereby exerting anti viral effect.  Found to be more effective than Lopinavir and Ritonavir in decreasing viral load in COVID-19 patients. |
| 6. | Nitazoxanide | Thiazolide compound with anti-bacterial, anti-viral and anti-parasitic properties.  It blocks the maturation of viral nucleocapsid N protein and thus impairs the production of viral particles in case of MERS-CoV. |
| 7. | Ivermectin | Broad spectrum anti-parasitic macrolide drug.  Functions by binding and impairing the cell transport proteins that essential for entry into the nucleus. |
| 8. | Corticosteroids | Exert immune-modulatory effects by inhibiting expression of genes encoding inflammatory molecules  Use of Corticosteroids in subjects with Acute respiratory distress syndrome (ARDS) was associated with reduced mortality.  Dexamethasone proven to be a life saving drug for severe COVID-19. |
| 9. | Tocilizumab and Sarilumab | Monoclonal antibody (MAb) antagonists of the IL6 receptor.  Drugs commonly used for treatment of rheumatoid arthritis.  Severe forms of COVID-19 are associated with elevated levels of IL6, causing acute respiratory distress syndrome (ARDS) even upon reduction of viral load.  These MAbs may play a vital role in reducing IL6 level and reduce instances ARDS in COVID-19 patients. |
| 10. | Bevacizumab | Monoclonal Antibody used to inhibit angiogenesis in cancer.  This MAb suppresses VEGF and reduces vascular permeability, thereby, decreasing amount of fluid entering lungs of COVID-19 patients with Acute respiratory distress syndrome (ARDS). |
| 11. | Fluvoxamine | Common anti depressant drug.  This serotonin re-uptake inhibitor may serve as an immune modulatory agent and shut down the inflammatory cascade from the endoplasmic reticulum by binding to the sigma-1 receptor |
| 12. | Remdesivir | Antiviral pro drug.  The active analogue of the pro drug inhibits the viral RNA dependent RNA polymerase (RdRp) and stopping viral replication.  Remdesivir also evades the proof reading mechanism (exoribonuclease) of Coronavirus. |
| 13. | Azithromycin | Broad spectrum macrolide antibiotic.  Used mainly for treatment of pulmonary, enteric and genitourinarytract infections. Acts as an acidotropic lipophilic weak base which modifies the pH of the endosome and trans-Golgi network and affects viral replication.  Interferes with viral entry by binding to viral spike (S) protein and humanreceptor protein ACE2 (angiotensin converting enzyme-2).  May exert interferon mediated anti viral immune response. |
| 14. | Baricitinib, Fedratinib, and Ruxolitinib | Potent JAK inhibitors selectively inhibiting JAK-STAT signaling.  Exerts anti-inflammatory effects.  Might be effective in controlling the cytokine storm in COVID-19.  Baricitinib is also predicted to hamper ACE2 mediated endocytosis. |
| 15. | Gimsilumab, Lenzilumab, Namilumab | These are anti-granulocyte-macrophage colony-stimulating factor (GM-CSF) antibodies. Blocks the inflammatory pathway in its early steps.  Being clinically tested for efficacy in COVID-19. |
| 16. | Thalidomide | Synthetic glutamic acid derivative.  Possess anti-inflammatory, anti-fibrotic, anti-angiogenesis, and immuno-modulatory effects.  Inhibits and downregulates COX2, PGE2, TNF-α, IL6 and IL1.  Used to treat severe H1N1 influenza-associated lung injury.  Being tested for its efficacy in treating cytokine storm and reducing lung injury and respiratory complications in COVID-19 |
| 17. | Favipiravir | Inhibits virus replication by binding and blocking the RdRp enzyme.  Its incorporation in RNA also terminates viral protein synthesis.  Classically used against influenza virus.  Also acts on SARS-CoV-2 replication; used for mild and moderate COVID cases. |
| 18. | APN01 | Soluble form of ACE2 delivered in high concentrations.  Could potentially block SARS-CoV-2 entry into target cells.  Under clinical trial. |

Note: Detailed Information have been collected from same set of references as mentioned in Table 3 in the main manuscript.

**Supplementary Table 7: List of medicinal plants with anti-viral, immune targeting and other medicinal properties.**

| **Sl.No.** | **Name of Plants** | **Type of anti viral or immune targeting effects exerted** |
| --- | --- | --- |
| 1. | Turmeric | Curcumin in turmeric is an immune-modulatory agent.  Has antibacterial, antiviral, anti-fungal, anti-inflammatory, anti-oxidant, anti-fibrotic, hepato-protective, anti-neoplastic and anti-tumor activities.  Reduces pro-inflammatory cytokines like IFN-γ, TNF-α, IL-1 and IL-8 via interaction with several signal transducers such as NF-κB, JAKs/STATSs, MAPKs and β-catenin.  Anti-inflammatory effects may be relevant in inflammatory respiratory diseases like COPD, asthma, acute respiratory distress syndrome (ARDS), pulmonary fibrosis, and acute lung injury. |
| 2. | Ashwagandha | Activates immune response.  Triggers Th1 cytokines and interferon expression.  Increases expression of co-stimulatory molecules and integrins. |
| 3. | Cinnamon | Have metal ion chelating, lipo-protective, anti-bacterial, anti-proliferative, radical scavenging activities.  Inhibits allergen specific immune responses.  Protects from systemic inflammation and lung injury by attenuating NLRP3 inflammasome activation. |
| 4. | Cardamom | Exerts anti-tumor effect by increasing the ability of NK cells to attack tumor cells.  Has anti-microbial activity.  Blocks i-NOS mediated NO generation and exerts anti-oxidant activity by restoring SOD, catalase and GSH levels.  Exerts anti-inflammatory effect by inhibiting mediators such as COX2, TNF-α and IL-6.  Mediates bronchodilatory effect in asthmatics. |
| 5. | Holy Basil | Have anti-microbial and anti-tussive activities.  Exerts anti-inflammatory effect by modulating cellular and humoral immunity.  Mediates anti oxidant activity by increasing glutathione peroxidase and catalase.  Elevates IFN-γ and IL-4.  Increases percentages of T-helper cells and NK-cells.  Traditionally used for cold and cough, bronchitis, bronchial asthma, hepatic injury, gastric ulcer. |
| 6. | Cumin | Thymoquinone in cumin has antioxidant, immuno-modulatory, anti-inflammatory, anti-microbial, anti-diabetic, anti-tumor, antihistaminic, anti-allergic, anti-tussive and bronchodilatory properties.  May be effective for allergic and obstructive respiratory disorders.  Suppresses inflammation by downregulation of COX2, IL-6, TNF-αand NO production, and enhancement of IL10 production.  Modulates cellular and humoral immunity.  Regulates Th1/Th2 immune response.  Enhances NK cell mediated cytotoxicity. |
| 7. | Neem | Exerts anti-inflammatory, antibacterial and antioxidant effects.  Attenuates release of pro-inflammatory cytokines such as TNF-α and IL-6, thus modulating immune response.  Inhibits monocyte chemoattractant protein-1 expression and recruitment of inflammatory cells.  Reduces iNOS level.  Mediates anti cancer effect by inhibiting cell proliferation and angiogenesis, maintaining redox balance and strengthening host immune response against tumor cells.  Neem extract enhances CD4^+^ Cell population in HIV/AIDS patients. |
| 8. | Saffron | Has anticonvulsant, antidepressant, anti-inflammatory, antitumor, radical scavenging and immuno-modulatory properties.  Exerts beneficial effects in asthma. |
| 9. | Amlaki | Has anti-diabetic, anti-microbial, anti-inflammatory, and immune-regulating activities.  Promotes NK cell activity and Antibody-dependent cellular cytotoxicity (ADCC).  Prevents apoptosis and DNA fragmentation.  Exerts radical scavenging activity, thus acting as an anti-oxidant. |
| 10. | Brahmi | Has anti-inflammatory, antidepressant, anti-microbial and hepato-protective properties.  Have immunomodulatory effects.  Exerts free radical scavenging and anti-oxidant activity.  Mediates anti-inflammatory effect by preventing the release of pro-inflammatory cytokines such as IL6, TNF-α from microglial cells and the immune cells of the brain.  Widely used from neurodegenerative and cognitive disorders. |
| 11. | Moringa | Activates CD8^+^ T cells, promotes IL-10, IL-2, IL-6 and TNF-α production.  Activates cellular immunity in Herpes Simplex Virus Type 1 mediated infection.  Confers protection against broncho-constriction and airway inflammation. |
| 12. | Liquorice Root  (Yashtimadhu) | Used as an anti-tussive and expectorant herbal medicine for cough and cold, sore throat.  Glycyrrhizin, the active compound of the liquorice root, inhibits SARS-associated coronavirus replication.  Glycyrrhizin also stimulates of interferon-gamma production by T cells, mediates immune-modulation and anti-inflammatory effect.  Reduces virus uptake by host cells (especially in case of influenza virus).  Exerts anti-inflammatory effect by inhibiting pro-inflammatory molecules like iNOS, COX2, IL-1β, TNF-α, IL-5 and IL-6 or by blocking transactivation of NF-κB.  Serves as natural remedy option for allergic asthma. |
| 13. | Shatavari | Serves as immuno-modulant, galactogauge, adaptogen, anti-tussive, anti-carcinogen, anti-oxidant, anti-diarrheal agent.  Modulates Th1/Th2 balance.  Promotes IgG secretion.  Inhibits IL-6 and promotes IL-12 production. |
| 14. | Coriander | Possess anti-bacterial and anti-fungal properties.  Have anti-oxidant and anti-inflammatory activities.  Boosts immunity. |
| 15. | Kapikacchu (Velvet Beans) | Modulates immune mediators such as NF-κB, IL-6, IFN-λ, TNF-α, IL-1β, iNOS and IL-2 in the central nervous system.  Used in neurological disorders.  Boosts innate immune response. |
| 16. | Ajwain | Has bronchodilatory effect and improves airflow to the lungs of asthmatics.  Possess anti-tussive effect and prevents coughing.  Possess anti-bacterial and anti-fungal properties.  Acts as anti spasmodic agent and anti-inflammatory agent. |
| 17. | Manjishtha | Have analgesic, anti-microbial and anti-inflammatory activities.  Serves as potential immune modulator and increases functions of the lymphatic system. |
| 18. | Bibhitaki | Is one of the important components of Triphala.  Have anti-oxidant, anti-microbial, anti-diarrheal, anti-tumor, anti-hypertensive, hepato-protective and antipyretic activities.  Relieves cold and cough.  Boosts immunity. |
| 19. | Guduchi, Giloy (Tinospora) | Serves as anti-oxidant, anti-hyperglycemic, antihyperlipidemic, hepato-protective, cardiovascular protective, neuroprotective, osteo-protective, radio-protective, anti-anxiety, adaptogenic agent, analgesic, anti-inflammatory, antipyretic, a thrombolytic agent, anti-diarrheal, anti-ulcer, anti-microbial and anti-cancer agent.  Regulates NF- кB signaling and production of pro-inflammatory mediators. May be effective for seasonal allergic rhinitis. |
| 20. | Haritaki | Is one of the components of Triphala.  Possess anti-fungal, anti-inflammatory, hypolipidemic and wound healing properties.  Beneficial in asthma, cough and cold. |
| 21. | Cinchona Bark | Source of chloroquine, a common anti-malarial drug; exerts an effect on SAR CoV-2 by immune modulation and blockage of viral entry. |
| 22. | Shatapushpa (Fennel) | Has apoptosis inducing activities.  Suppresses immune response.  Have potential for treating airway inflammation in acute lung injury by regulating Th17 and Treg immune response. |
| 23. | Triphala | Comprises of Amlaki, Bibhitaki, Haritaki.  Possess anti-microbial activity, anti-aging property, anti-oxidant activity and anti-neoplastic activity.  Exerts anti-inflammatory effect via decreased expression of inflammatory mediators such as IL-17, COX-2, iNOS, TNF-α, IL-1β, VEGF, IL-6 and RANKL through inhibition of NF-κB activation. |
| 24. | Jatiphala (Nutmeg) | Has immuno-modulatory function.  Macelignan in nutmeg has anti-inflammatory property.  Macelignan inhibits Th2 cytokines such as IL-4.  Macelignan attenuates eosinophilic airway inflammation and airway hyper-responsiveness in allergic asthma. |
| 25. | Jatamansi | Have anti-oxidant activity.  Also have anti-inflammatory, immuno-modulatory and wound-healing properties. |
| 26. | Vidanga | Used for treating tumors, ascites, bronchitis, jaundice, heart and brain diseases in traditional Indian medicine.  Effective against acute respiratory distress syndrome (ARDS) due to its anti-inflammatory property.  Ameliorates pro-inflammatory cytokines and suppresses TNF-α production.  Have anti-oxidant, anti-tumor, analgesic, anticonvulsant, anti-fertility, wound healing, cardio-protective properties. |
| 27. | Gokshura (Tribulus) | Used as a cardiotonic, diuretic, and aphrodisiac.  Has reno-protecctive effect.  Can reduce inflammation and fibrosis in the lungs by lowering the expression of TNF-α, IL-6, IL-8 and TGFβ1. |
| 28. | Bhringaraj (Eclipta) | Exhibit promising pharmacological properties, like hepato-protective, osteo-protective, cytotoxic, hypoglycaemic, anti-inflammatory, anti-microbial, hypolipidemic, rejuvenative and neuro-protective effects.  Exerts anti-inflammatory effect via regulation of NF-κB pathway and pro-inflammatory mediators. |
| 29. | Punarnava (Boerhavia) | Possess anti-microbial, anti-diabetic, anti-fibrolytic, anti-asthmatic, spasmolytic, anti-convulsant, anti-cancer and hepato-protective activities.  Quenches free radicals, thus, acting as an anti-oxidant.  Have anti-inflammatory properties.  Punarnavine, an alkaloid in Boerhavia exerts immuno-modulatory activities by reducing TNF-α, IL-1β, IL-6 production and by increasing titer of circulating antibody. |
| 30. | Bhunimba (Andrographis) | Has several biological activities like antioxidant, anti-cancer, anti-inflammatory, immuno-modulatory, antiseptic, antimicrobial, cytotoxic, hypolipidemic, cardio-protective, hepato-protective, and neuro-protective effects.  Provides symptomatic relief of acute respiratory tract infections.  Exhibits anti-viral activity against cold and influenza virus. |
| 31. | Shankha pushpi (Dwarf morning glory) | Possess anti-allergy, anti-tussive and anti-asthmatic activities.  Used in the treatment of respiratory disorders including bronchitis, asthma.  Has anti-arthritic activity.  Possess antibacterial activity (specifically against Gram-negative bacteria) and prominent immuno-modulating activity. |
| 32. | Vidari (Indian Kudzu) | Serves as an immune booster.  Acts as an anti-oxidant with potent superoxide and hydroxyl radical scavenging activities.  Serves as an anti-inflammatory agent by inhibiting inflammatory mediators such as CRP, NF-κB, COX-2, iNOS, IL-1β, IL-6 and TNF-α.  Has immuno-modulatory potential.  Possess anti-mycobacterial activity. |

Note: Detailed Information have been collected from same set of references as mentioned in Table 5 in the main manuscript.
